# An inherited TBX3 alteration in a prenatal case of ulnar‐mammary syndrome: Clinical assessment and functional characterization in *Drosophila melanogaster*

**DOI:** 10.1002/jcp.31440

**Published:** 2024-09-25

**Authors:** Irene Bottillo, Andrea D'Alessandro, Maria Pia Ciccone, Gianluca Cestra, Gianluca Di Giacomo, Evelina Silvestri, Marco Castori, Francesco Brancati, Andrea Lenzi, Alessandro Paiardini, Silvia Majore, Giovanni Cenci, Paola Grammatico

**Affiliations:** ^1^ Division of Medical Genetics, Department of Experimental Medicine, San Camillo‐Forlanini Hospital Sapienza University Rome Italy; ^2^ Department of Biology and Biotechnologies “C. Darwin” Sapienza University of Rome Rome Italy; ^3^ Istituto Pasteur Italia‐Fondazione Cenci Bolognetti Rome Italy; ^4^ Institute of Molecular Biology and Pathology (IBPM), National Research Council (CNR) Rome Italy; ^5^ Fondazione Santa Lucia IRCCS, c/o CERC Rome Italy; ^6^ Unit of Fetal and Neonatal Pathology, Division of Pathology San Camillo‐Forlanini Hospital Rome Italy; ^7^ Division of Medical Genetics Fondazione IRCCS‐Casa Sollievo della Sofferenza San Giovanni Rotondo Italy; ^8^ Human Genetics Laboratory, Department of Life, Health and Environmental Sciences University of L'Aquila Italy; ^9^ San Raffaele Roma IRCCS Rome Italy; ^10^ Department of Experimental Medicine Sapienza University of Rome Rome Italy; ^11^ Department of Biochemical Sciences “A. Rossi Fanelli” Sapienza University of Rome Rome Italy

**Keywords:** Drosophila development, Humanized Drosophila, TBX3, Ulnar‐mammary syndrome

## Abstract

Ulnar mammary syndrome (UMS) results from heterozygous variants in the *TBX3* gene and impacts limb, tooth, hair, apocrine gland, and genitalia development. The expressivity of UMS is highly variable with no established genotype–phenotype correlations. *TBX3* belongs to the *Tbx* gene family, which encodes transcription factors characterized by the presence of a T‐box DNA‐binding domain. We describe a fetus exhibiting severe upper limb defects and harboring the novel *TBX3*:c.400 C > T (p.P134S) variant inherited from the mother who remained clinically misdiagnosed until prenatal diagnosis. Literature revision was conducted to uncover the *TBX3* clinical and mutational spectrum. Moreover, we generated a *Drosophila* humanized model for *TBX3* to study the developmental consequences of the p.P134S as well as of other variants targeting different regions of the protein.

Phenotypic analysis in flies, coupled with *in silico* modeling on the *TBX3* variants, suggested that the c.400 C > T is UMS‐causing and impacts TBX3 localization. Comparative analyses of the fly phenotypes caused by the expression of all variants, demonstrated that missense changes in the T‐box domain affect more significantly TBX3 activity than variants outside this domain. To improve the clinicians' recognition of UMS, we estimated the frequency of the main clinical features of the disease. Core features often present pre‐pubertally include defects of the ulna and/or of ulnar ray, hypoplastic nipples and/or areolas and, less frequently, genitalia anomalies in young males. These results enhance our understanding of the molecular basis and the clinical spectrum of UMS, shedding light on the functional consequences of *TBX3* variants in a developmental context.

## 1 INTRODUCTION

Ulnar‐mammary syndrome (UMS, OMIM #181450), also known as Shinzel syndrome, is a rare autosomal dominant disorder mainly characterized by ulnar ray defects, underdevelopment of the mammary and apocrine glands, and diminished axillary hair (Joss et al., 2011). Additional findings may include hormonal deficiencies, delayed puberty (particularly in males), short stature, obesity, genitalia abnormalities, teeth anomalies, anal atresia/stenosis, ventricular septal defect, heart conduction defects and facial features such as wide face, prominent chin and broad nose (Joss et al., 2011).

Deleterious variants in the T‐box 3 gene *TBX3* have been associated with UMS in humans (Bamshad et al., 1996). TBX3 is a transcription factor member of a phylogenetically conserved gene family that shares the T‐box DNA‐binding domain and regulates the development of the heart, lung, mammary gland, limb, and digits (Khan et al., 2020). Additionally, *TBX3* coding sequence includes two repression domains (R1 and R2), an activation domain (A) and a nuclear localization signal (NLS). *TBX3* has been characterized by four distinct transcript variants, with TBX3 and TBX3 + 2a being the most prevalent ones (Bamshad et al., 1996; W. Fan et al., 2004). Pleiotropy of UMS is indicative of the complex transcriptional networks in which TBX3 participates during development and haploinsufficiency is considered the prevalent disease mechanism. To date, not any strong genotype–phenotype correlations exist and variable expressivity is a main feature of MS (Washkowitz et al., 2012).

Alterations of *TBX3* have been associated with developmental defects in *Drosophila melanogaster* (del Álamo Rodríguez et al., 2004), *D. rerio* (Ribeiro et al., 2007), and *Mus musculus* (Davenport et al., 2003) suggesting an evolutionarily conserved role for this transcription factor in the development of organ systems. Yet, mice lacking *Tbx3* are not viable and display a wide range of onset of early lethality, between E10.5 and E16.5 (Davenport et al., 2003). Human TBX3 shares a high level of similarity (~50%) with the *Drosophila bifid(bi)/optomotor‐blind (omb)* gene product, which controls cell proliferation, viability and cell migration of wing and eye discs (Z. Fan et al., 2021). The folding of human TBX3 and *Drosophila* Omb proteins is also highly similar and there is a specific correspondence in some conserved amino acids that directly contact DNA (Coll et al., 2002). This suggests that *Drosophila* could be exploited as a system model to genetically dissect the TBX3‐dependent pathways.

We describe the private heterozygous *TBX3* c.400 C > T (p.P134S) variant in a fetus with a severe reduction of upper limbs. The c.400 C > T change resulted to be inherited from the affected mother, who remained unaware of being affected since the identification of the fetal anomalies. To understand the effects of the p.P134S alteration at an organismal level and its relevance to the UMS, we have established the first *Drosophila* humanized model for *TBX3* by generating transgenic flies that express the p.P134S, as well as other *TBX3* variants targeting different regions of the protein. Moreover, the revision of literature allowed us to improve the definition of the *TBX3* constitutional mutational *spectrum* and the prevalence of the main clinical signs associated with UMS.

## 2 RESULTS

### 2.1 Clinical and genetic studies of the family

A 33‐year‐old woman requested consultation at the genetic unit of the San Camillo Hospital, Sapienza University of Rome, at 16 weeks of her first pregnancy due to fetal anomalies at prenatal ultrasound scan. Pregnancy was obtained by natural conception after 2 years of attempts. A maternal febrile episode lasting about 4 days occurred in the first trimester. Maternal diabetes and exposure to known hexogen teratogens were denied. Noninvasive Prenatal Test (NIPT), performed elsewhere at 12 weeks of gestation, showed a low risk for fetal chromosome aneuploidies. Obstetric ultrasound at 15th + 5 weeks demonstrated severe reduction defects involving forearms and hands bilaterally, apparently in the absence of any other structural anomalies.

**Figure 1:**
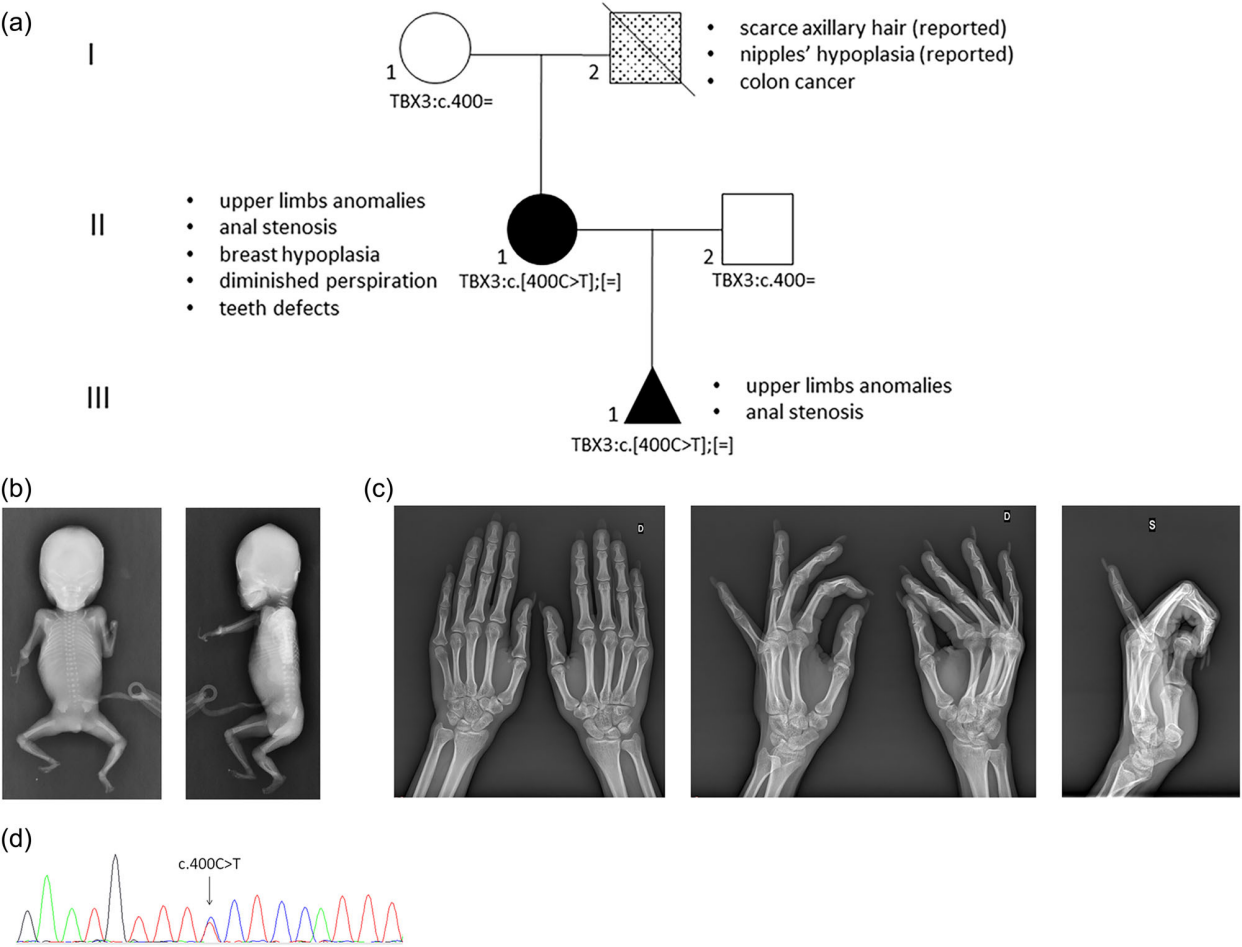
Clinical, molecular and radiological characterization of the pedigree. (a) Family pedigree. The phenotype of each patient is shown with different filling motifs and the clinical legend is given alongside each patient. The *TBX3* genotypes are given under each case's symbol. (b) Frontal and lateral views of the fetus showing absence of the left hand and a right hand with only two ossified digits, forearms composed of a single long bone, presumably the radius, which was severely shortened and bowed on the right. (c) X‐rays of the proband's hands showing absence of the pisiform and hypoplasia of the trapezius, hypoplastic fifth and fourth metacarpal bones, undertubulation of the fifth metacarpus which appeared slightly bowed, overtubulation of the proximal phalanx, and hypoplasia of the distal phalanx and ungual tuft of the fifth finger, on the left. Right hand was normal. (d) The Sanger sequencing electropherogram from the fetus showing the presence of the *TBX3:*c.400 C > T in heterozygosity.

At first evaluation, the woman appeared healthy but referred to be born with a minor malformation of her left fifth finger that appeared to be slightly hypoplastic. Family history offered further insights. In fact, her father, who died at 58 years due to colon cancer, was described with unilateral abnormalities of the hand, presumably consisting of unilateral mild hypoplasia and incomplete duplication of the 5th finger (Figure 1a). Amniocentesis was offered with the purpose of performing karyotyping and exome sequencing (ES) to improve prenatal counselling. After multidisciplinary consultations at the 17th + 1 week of gestation, the woman and her husband decided to medically terminate the pregnancy before genetic results.

Fetal autopsy confirmed the presence of severe reduction defects of the upper limbs. The forearms and extremities appeared asymmetrically hypoplastic/absent with a more severe involvement on the right. Additional findings included anal stenosis and minor facial dysmorphisms. Post‐termination fetal X‐rays confirmed the absence of the left hand and a right hand with only two ossified digits. Both forearms seemed composed of a single long bone, presumably the radius, which was severely shortened and bowed on the right (Figure 1b). ES analysis performed on DNA extracted from a small fragment of fetal skin, failed due to lack of sequencing coverage.

At follow‐up, it emerged that the woman was born with an imperforate anus, which was surgically repaired. In addition, she underwent additive mastoplasty due to severe mammary hypoplasia in adulthood. She also reported diminished perspiration and body odor, and poor axillary hair; features suggestive of defective development of the skin adnexa. Menarche occurred at age 15 years and menstrual cycles were always scarce. Hands X‐rays examination showed absence of the pisiform and hypoplasia of the trapezius, hypoplastic fifth and fourth metacarpal bones, undertubulation of the fifth metacarpus which appeared slightly bowed, overtubulation of the proximal phalanx, and hypoplasia of the distal phalanx and ungual tuft of the fifth finger, on the left (Figure 1c). The right hand was unremarkable. Endocrinological screening, long strip ECG and echocardiogram yielded negative results. The proband also reported that the father had scantly axillary hair and nipple hypoplasia.

ES analysis focused on genes involved in limb development and congenital anomalies revealed that the woman was carrier of the *TBX3* NM_005996.4:c.400 C > T (p.P134S) variant at the heterozygous state. Segregation analysis in the extended family demonstrated that the variant was present in the affected fetus (Figure 1d) and absent in the unaffected proband's mother. This variant was considered rare because absent in GnomAD 2.1 population database, fell in a key functional domain (T‐box domain) and was predicted deleterious by *in silico* tools (REVEL score 0.968). Reverse phenotyping of the family allowed to recognize in the anomalies observed in the proband and fetus the main features of UMS. Accordingly, the variant was provisionally classified as likely pathogenic (PM2_Moderate, PM1_Moderate, PP3_Supporting, PP4_Supporting).

The clinical, molecular and radiological findings of the family members are presented in Figure 1.

### 2.2 Literature revision on patients carrying a *TBX3* alteration

The literature was reviewed to collect all constitutional *TBX3* variants that have been so far described in UMS cases. We queried PubMed using the following strings: [TBX3 and UMS] and [TBX3 and Ulnar‐Mammary syndrome] with no restriction on publication date. All studies that described the clinical features of patients carrying a molecularly defined *TBX3* variant were considered. The revision included the following publications: (Al‐Qattan et al., 2020; Bamshad et al., 1999; Galazzi et al., 2018; Joss et al., 2011; Meneghini et al., 2006; Sasaki et al., 2002; Tanteles et al., 2017; Tung et al., 2022; Wollnik et al., 2002); this report. We have also integrated all the *TBX3* variants reported in the ClinVar database (https://www.ncbi.nlm.nih.gov/clinvar/).

The clinical findings were summarized based on their association with the respective variants (Supplementary Material 1). According to our literature revision, 28 different pathogenic/likely pathogenic *TBX3* variants were documented. These variants were found in 87 UMS patients described postnatally. To our knowledge, the fetus described in this work represents the first documentation of UMS in a prenatal setting. Among the 87 cases, 5 cases were reported in ClinVar with no specific clinical description. Out of the 28 pathogenic/likely pathogenic *TBX3* variants, 19 (68%) were reported in familial cases.

**Figure 2:**
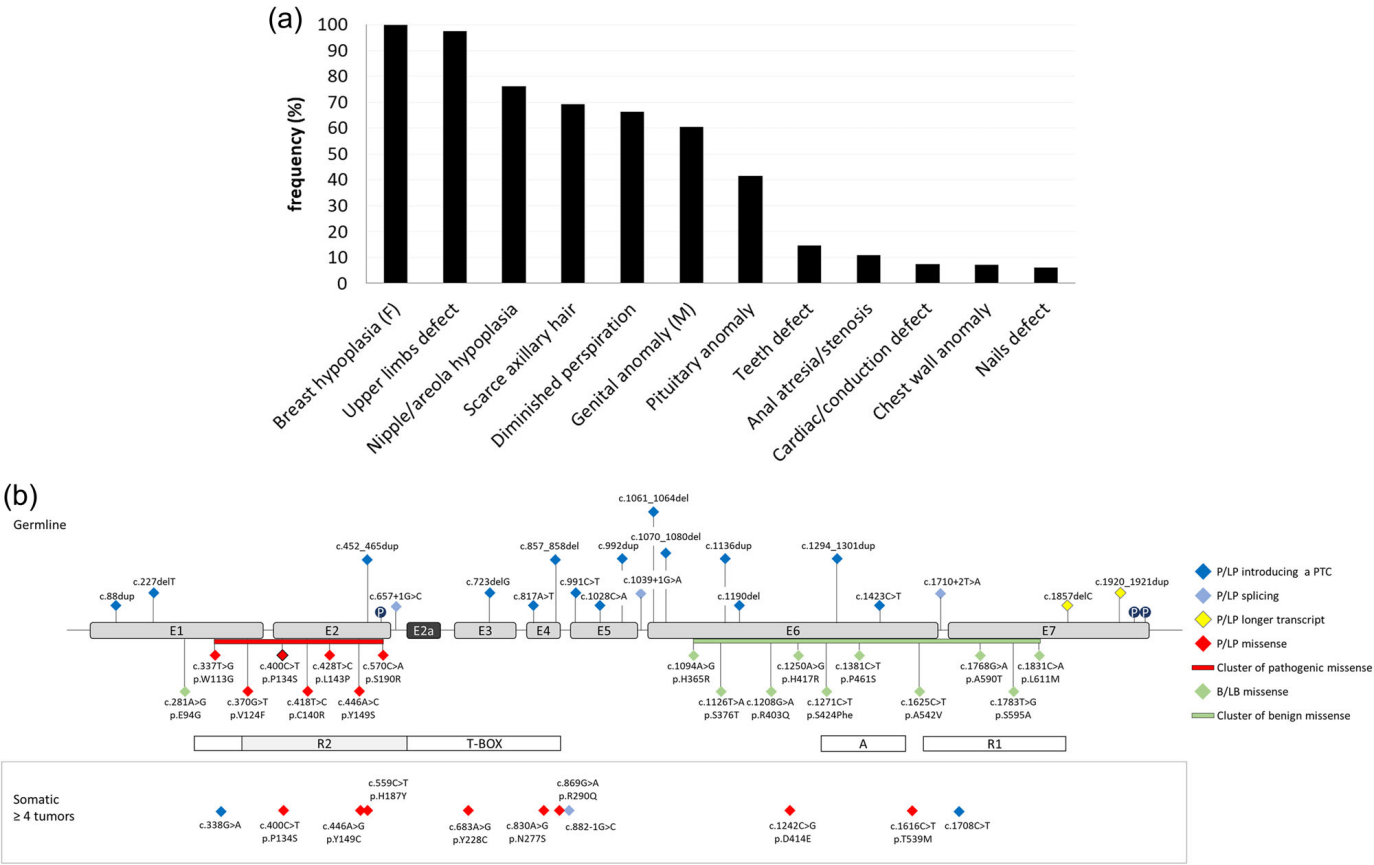
Clinical and molecular revision of the literature. (a) Clinical revision of the literature**.** Frequency (y‐axis) of the clinical features (x‐axis) reported for UMS patients. Clinical signs restricted to one sex are indicated with F (females) or M (males). (b) Schematic structure of *TBX3* gene and known mutations associated to UMS. Exons are in scale, introns are not in scale. The protein's functional domains are shown as boxes on the bottom of the exons. The nomenclature of each genomic change is given according to NM_005996.4. E: exon; P: phosphorylation site; P/LP: pathogenic/likely pathogenic; B/LB: benign/likely benign.

Figure 2a shows the prevalence of each clinical feature reported in UMS patients. The clinical signs present in more than 50% of the cases included: defects of the upper limbs (i.e., ulna absence/anomaly, radius bowing, humerus shortening), breast and nipple hypoplasia (respectively in females and in both sexes), scarce axillary hair, diminished perspiration and males' genitalia anomalies.

About half (54%) of the *TBX3* constitutional mutational spectrum consisted of truncating variants distributed along the entire gene coding sequence up to its penultimate exon. Missense pathogenic/likely pathogenic variants accounted for about 25% of the mutational spectrum and all mapped in the T‐box domain, particularly in the R2 region. In contrast, the majority of the constitutional benign or likely benign missense variants extracted from the ClinVar database were located within the A and R1 domains (Figure 2b). Finally, the *C*atalogue *O*f *S*omatic *M*utations *I*n *C*ancer (COSMIC) database (https://cancer.sanger.ac.uk/cosmic) was accessed to retrieve the most frequent somatic *TBX3* alterations identified at least on 4 different tumor samples (Figure 2a). Most of these somatic variants were missense and mapped in the T‐box. The c.400 C > T (p.P134S) was also annotated in the *TBX3* somatic mutational *spectrum* (COSMIC mutation ID: COSM5196199).≥

To assess the functional consequences of the p.P134S variant and its relevance to UMS, we exploited the well‐established in vivo system model *D. melanogaster* to express this mutant protein in different tissues and organs. In addition, we performed an *in silico* characterization and protein dynamic simulations of the p.P134S. For a comparative analysis we also modeled three additional *TBX3* variants, namely the p.N277S, the p.T539M and the p.E695R, that were selected along the gene's mutational spectrum. The p.N277S and the p.T539M map in the T‐box and in the A domain respectively and were annotated in COSMIC tumor samples. However, they were never reported to be associated with UMS at constitutional level. The p.E695R is a missense change that is predicted to result from the shifts introduced by both the c.1857delC and the c.1920_1921dup at the very 3' end of the R1 encoding sequence.

### 2.3 Heterologous overexpression of wild‐type and mutant human TBX3 proteins in flies

**Figure 3:**
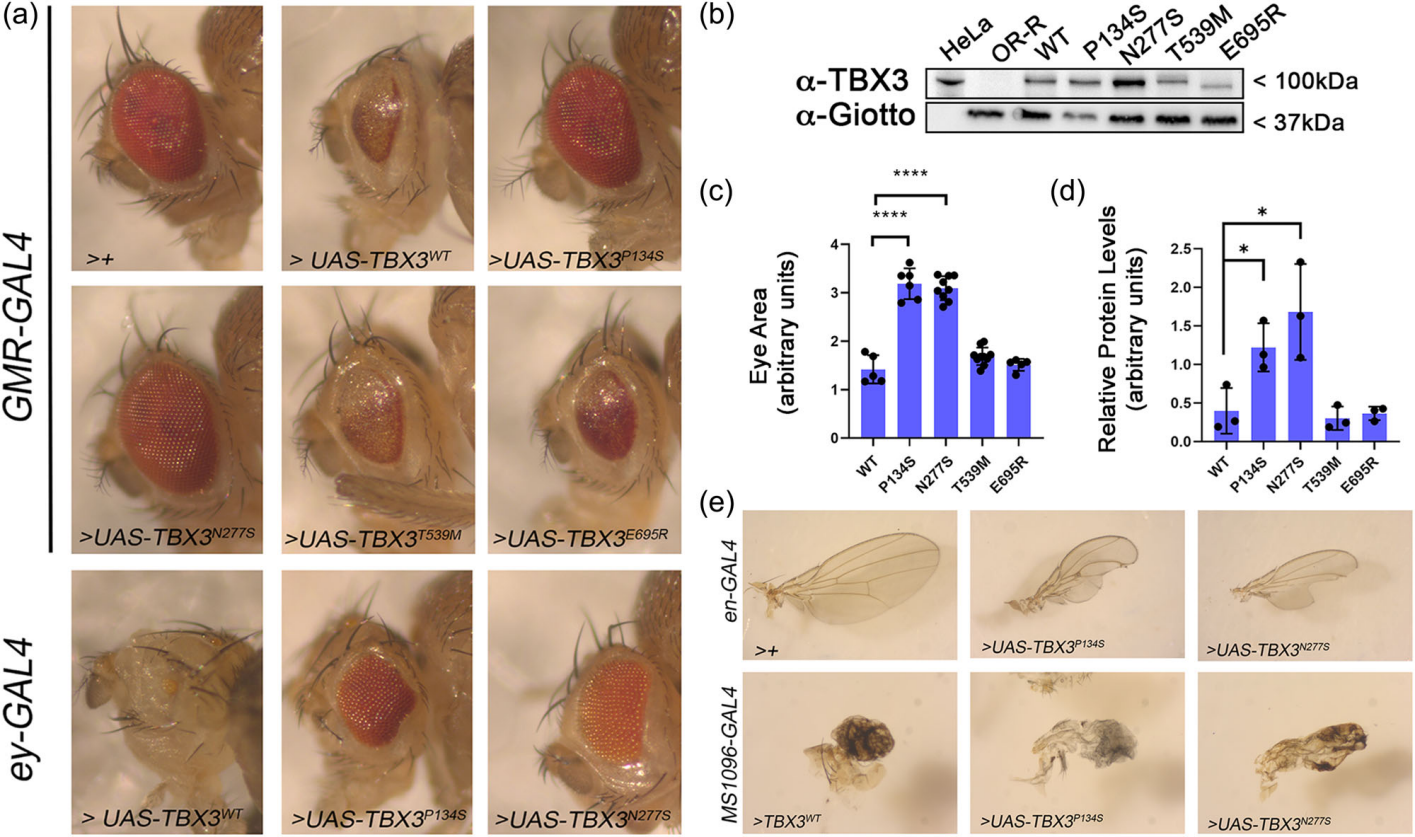
Ectopic expression of wild‐type and mutant human *TBX3* transgenes affects the development of eyes and wings in Drosophila. (a) Eyes images were taken from the progeny of crosses between the *ey‐GAL4* the *GMR‐GAL4* drivers and the different *UAS‐TBX3* transgenes encoding the reference TBX3 and selected variants at 18°C and 25°C, respectively (see also Table 1). Note that the rare viable escaper *ey ‐GAL4* > *UAS‐TBX3* ^*WT*^ flies show almost total loss of the eye. *GAL4 >+* refers to the internal control. (b) Western Blot from *GMR‐GAL4* > *UAS‐TBX3* heads indicated in A. human HeLa cell extracts and wild‐type Oregon R heads were used as positive and negative controls, respectively of the TBX3 expression. Anti‐Giotto antibody was used as loading control. (c) Quantification of eye area of *GMR‐GAL4* > *UAS‐TBX3* flies shown in A (****p < 0.001; Student *t*‐test). (d) Quantification of TBX3 levels from at least 4 different WBs as in C. Error bars represent the standard errors of the mean. (*p < 0.05; Student t‐test). (e) Wings were dissected from adult progeny of crosses between *en‐GAL4 or MS1096‐GAL*4 and the different *UAS‐TBX3* transgenic flies expressing the reference TBX3 and selected variants proteins at 18°C (see also Table 1). Note that the rare viable escaper *MS1096‐GAL4* > *UAS‐TBX3* ^*WT*^ fly shows severe wing development defects similar to the *TBX3* ^*P134S*^ and *TBX3* ^*N277S*^ viable flies.

We generated five *Drosophila* transgenic lines expressing, under the *UAS* promoter, the human reference *TBX3* gene (hereafter *UAS‐TBX3* ^*WT*^), as well as the variants *TBX3* c400C>T, *TBX3* c800A>G, *tbx3* c.1616 C > T and *TBX3* c.2782‐2783insA (hereafter *UAS‐TBX3* ^*P134S*^, *UAS‐TBX3* ^*N277S*^, *UAS‐TBX3* ^*T539M*^ and *UAS‐TBX3* ^*E695R*^, Supplementary Material 2). To overcome potential position effect‐dependent differences in the expression of these variants, all constructs were inserted in the same chromosomal location of chromosome 2 exploiting the PhiC31 integrase system (see Methods). To evaluate the functional significance of each variant, we leveraged the *Drosophila* UASXGAL4 binary system to carry out expression studies using different tissue specific GAL4 drivers (Caygill & Brand, 2016) (Table 1). The ubiquitous expression of WT or mutant *TBX3* variants, driven by the *GAL4* transcription factor under the control of *Tubulin* enhancer caused lethality during the third instar larvae‐pupae transition suggesting the flies are highly sensitive to the dosage of the human TBX3 irrespective to the variants. This result was not unexpected as similar effects were previously described for TBX2 (Liu et al., 2018). Next, we drove tissue‐specific expression of TBX3 wild‐type and mutant proteins using the *eyeless (ey*) ‐GAL4 and *Glass Multimer Report (GMR)‐GAL 4* drivers in the eye imaginal discs, a well‐established model for organ growth and cell proliferation (Weasner & Kumar, 2022). The phenotypic characterization of flies expressing these transgenes in the eye revealed that their ectopic expression caused different degrees of toxicity. In particular, the *eyGAL4*‐driven expression of TBX3^WT^, TBX3^T539M^ and TBX3^E695R^ early during eye disc development, before furrow formation (Bonini et al., 1997), caused either lethality at 25°C or rare viable escapers with strongly reduced or absent eyes at 18°C (Figure 3a). The differences observed in the phenotypic penetrance of each allele at different growing temperatures are consistent with the expression profile of transgenes in fly, which significantly increases with the rise of temperatures. Flies expressing the transgenes under *GMR*‐*GAL4*, which drives the expression later during eye disc development in the cells posterior to the morphogenetic furrow of the eye discs (Freeman, 1996; Sawado et al., 1998), were viable at both temperatures although adult eyes elicited highly degenerated ommatidia (Figure 3a). Interestingly, flies expressing TBX3^WT^ or any other variant carrying an intact T‐box domain, such as TBX3^T539M^ and TBX3^E695R^, show significantly more severe phenotypes than *TBX3* ^*P134S*^ *‐* and *TBX3* ^*N277S*^‐expressing flies, which harbor alterations in the same domain. This strongly suggests that the ectopic expression of TBX3 proteins that are still able to bind the DNA through a functional T‐box domain significantly hampers the *Drosophila* developmental program. Moreover, the observation that the eye phenotype of *ey ‐GAL4* > *UAS‐TBX3* ^*P134S*^, *ey‐GAL4* > *UAS‐TBX3* ^*N277S*^, *GMR ‐GAL4* > *UAS‐TBX3* ^*P134S*^ and *GMR‐ GAL4* > *UAS‐TBX3* ^*N277S*^ adults was less severe than other variants (Figure 3a, c), suggests that *TBX3* ^*P134S*^ or *TBX3* ^*N277S*^ could represent partial loss of function TBX3 alleles. In addition, WB analyses on protein extracts from wild‐type and mutant *GMR‐GAL4* > *UAS TBX3* expressing adult heads, revealed that TBX3^WT^, TBX3^T539M^ and TBX3^E695R^ protein levels are not more abundant than those of TBX3^P134S^ and TBX3^N277S^ ruling out the possibility that the severity of eye phenotypes is associated with a high expression of these variants (Figure 3b, d; Supplementary Figure 1).

**Table 1:** Classification of developmental defects in *TBX3*‐expressing flies. All phenotypes were scored in the progeny of crosses between flies expressing the *GAL4* drivers and each *UAS‐TBX3* transgene at either 18°C or 25°C. Crosses with the *Tubulin ‐GAL4GAL80* drivers were maintained at 18°C for 5 days (to allow the expression of *GAL80* that in turn represses Gal4) and then shifted to 29°C for 2 more additional days to inhibit Gal80 thus activating the Gal4‐induced expression. At least 30 adults were analyzed for each cross (LL: Late Lethal; EL: Early Lethal; SL: semi lethal; PL: Pupal Lethal: AW: Aberrant Wing blade; IE: Irregular Eye Ommatidia; RE: Normal Eyes; *: Rare viable escapers; **: *Tubulin‐GAL4GAL80*.

		UAS‐TBX3 Mutants				
GAL4 Drivers	Temp	*WT*	*P134S*	*N277S*	*T539M*	*E695R*
*engrailed*	18°C	EL	AW	AW	LL	EL
	25°C	EL	EL	AW	EL	EL
*MS1096*	18°C	LL*	AW	AW	LL*	LL*
	25°C	LL	AW	AW	LL	LL
*eyeless*	18°C	LL*	IE	IE	LL*	LL
	25°C	LL	IE	IE	LL	LL
*GMR*	18°C	IE	RE	RE	IE	IE
	25°C	IE	RE	RE	IE	IE
*69B*	18°C	EL	SL	LL	EL	EL
	25°C	EL	SL	LL	EL	EL
Tubulin**	18°C > 29°C	LL	SL	PL	LL	LL

We also assessed the effect TBX3 wild‐type and mutant protein ectopic expression under the control of *engrailed (en)‐* or *MS1096‐GAL4*, which drive their expression in the wing imaginal discs, a well‐characterized model for vertebrate limb development (Johnson & Tabin, 1997; Klein, 2001). The *en‐* and *MS1096*‐driven specific expression in wing disc posterior compartment and pouch, respectively (Li et al., 2013; Rintelen et al., 2003), of TBX3^WT^, TBX3^T539M^ and TBX3^E695R^ was overall lethal, although rare adult escapers with strongly aborted wing development were seldom found (Figure 3e, Table 1). Yet, the *MS1096*‐*GAL4* driven expression of *UAS‐TBX3* ^*P134S*^ and *UAS‐TBX3* ^*N277S*^ was viable and yielded severe development of adult wings at both 18°C and 25°C growing temperatures. Finally, the *engrailed*‐ GAL‐ driven expression of *UAS‐TBX3* ^*P134S*^ and *UAS‐TBX3* ^*N277S*^ resulted in viable adults with abnormal wings at 18°C. Yet, at 25°C the same expression was lethal for TBX3^P134S^ but not for the TBX3^N277S^‐expressing flies which in contrast were viable and elicited a strong wings phenotype (Figure 3e, Table 1). Overall, these observations on the different degrees of toxicity of wild‐type and mutant TBX3 proteins are in line with the characterization of the eye phenotype and confirm that TBX3^P134S^ and TBX3^N277S^ encoding sequences behave at least as partial loss of function alleles. Moreover, the reduced toxicity of TBX3^N277S^ compared to TBX3^P134S^, strongly suggests that TBX3^N277S^ alteration may hamper the transcriptional activity of the T‐box domain even more significantly than TBX3^P134S^.

**Figure 4:**
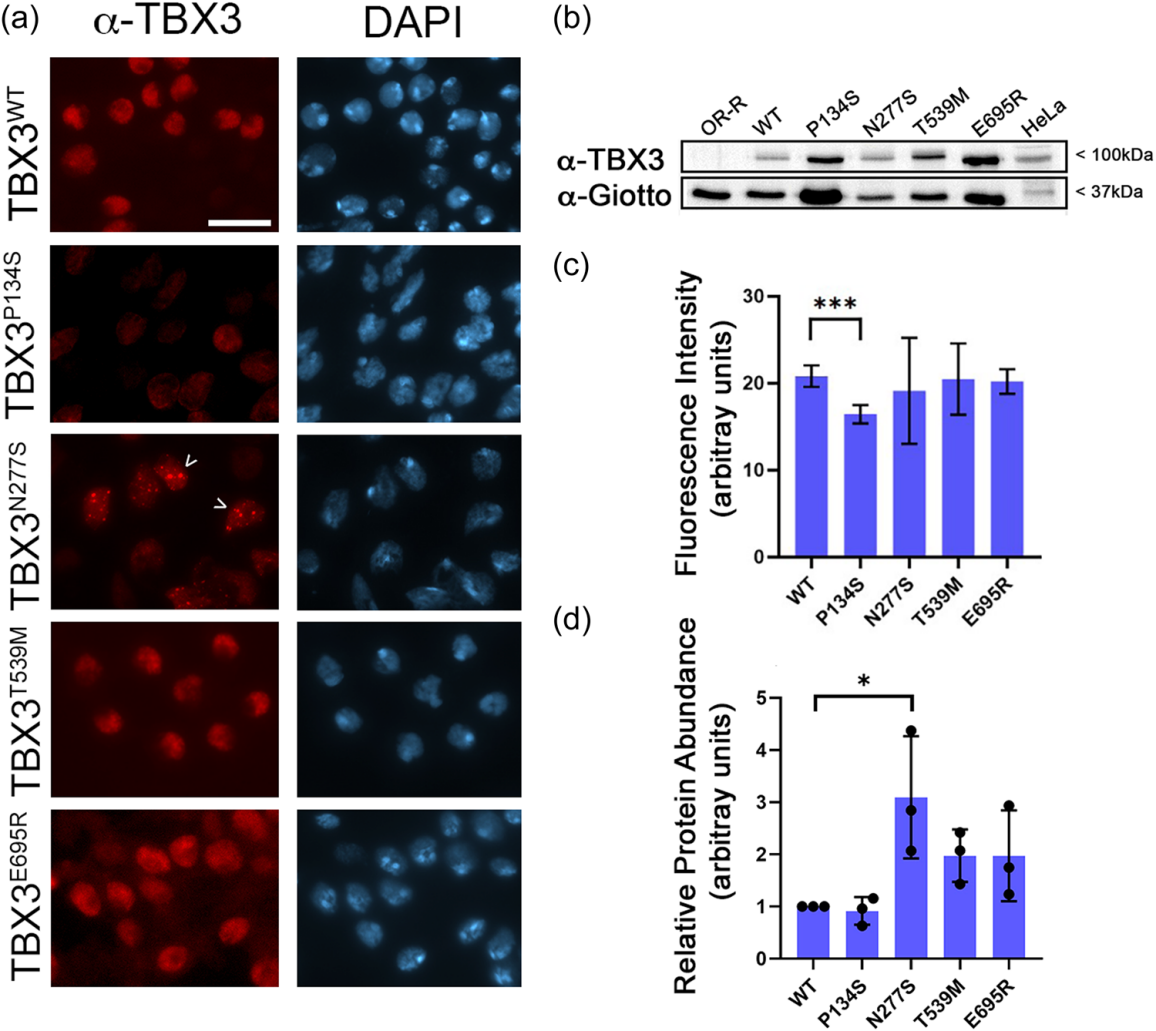
Localization of wild‐type and mutant TBX3 proteins in larval neuroblasts. (a) *Tubulin‐GAL4GAL80* > *UAS‐TBX3* expressing larval neuroblasts stained with anti‐TBX3 (red) and DAPI (blue; for DNA). Note that TBX3^P134S^ and TBX3^N277S^ show a nuclear localization pattern different from the wild‐type and TBX3^T539M^ and TBX3^E695S^ that display a similar subcellular localization. Arrow heads indicate TBX3^N277S^ aggregates. See Text for further details. Scale Bar: 10μm. (b) Western blot from larval brain extracts of the same TBX3‐expressing flies shown in A. anti‐Giotto has been used as a control. (c) Quantification of TBX3 fluorescence. At least 150 nuclei for each genotype were analyzed. (d) Quantification of protein levels from at least 3 different WBs. Error bars represent the standard errors of the mean. (*p < 0.05; Student t‐test).

To further understand the different degree of toxicity of all *TBX3* variants, we investigated the subcellular localization of these proteins in larval brains that are considered one of the best fly organ/tissue for protein localization during cell division (Gallaud et al., 2017; Keegan & Hughes, 2021). Since adult lethality associated with the ectopic expression of the transgenes was observed primarily at adult stages, we carried out immunofluorescence experiments on neuroblasts from third‐instar larvae brains. Anti‐TBX3 staining of *Tubulin‐GAL4* > *UAS‐ TBX3* ^*WT*^‐expressing brains revealed that this protein strongly localizes on chromatin in the ~90% of larval brain cells (N = 150) (Figure 4). This finding, which is consistent with previous localization analyses of the human protein (Carlson, 2001; Willmer et al., 2015), confirms that TBX3 behaves as a nuclear factor also when expressed in flies. A similar localization pattern was also found for TBX3^T539M^ and TBX3^E695R^ variants indicating that both missense changes do not affect the nuclear localization of the protein (Figure 4). In contrast, immunostaining of TBX3^P134S^ revealed a moderate, still statistically significant, reduction in the nuclear localization of TBX3 suggesting that c.400 C > T missense variant, although it does not affect the expression levels of the variant compared to TBX3^WT^, TBX3^T539M^ and TBX3^E695R^ (Figure 4d), likely impairs its binding to chromatin. Finally, we found that the expression of TBX3^N277S^ resulted in the formation of TBX3 nuclear puncta (~45%; *N* = 150) suggesting that this variant induced the formation of potential aggregates. The formation of these aggregates was even more evident by IF on polytene chromosomes (Supplementary Figure 2), that also confirmed that both TBX3^P134S^ and TBX3^N277S^ retain a very limited ability to bind to chromatin. However, a comparative localization analysis of the subcellular localization of TBX3^WT^, TBX3^T539M^ and TBX3^E695R^ variants on polytene chromosomes was not possible, as the expression of these latter impaired salivary gland development. Surprisingly, we noticed that protein levels of TBX3^N277S^ (Figure 4b and Supplementary Figure 3), but not its corresponding RNA (Supplementary Figure 4), in larval brains were more abundant than both the wild‐type and the other TBX3 variants, indicating that the c.803 A > G substitution may affect the turnover of the protein through still‐uncharacterized mechanisms. However, our WB results from larval brain extracts (Figure 4b, d) support the view that the different degree of lethality and/or toxicity of TBX3 proteins does not correlate with their abundance.

### 2.4 Molecular modeling for the analysis of protein structures

**Figure 5:**
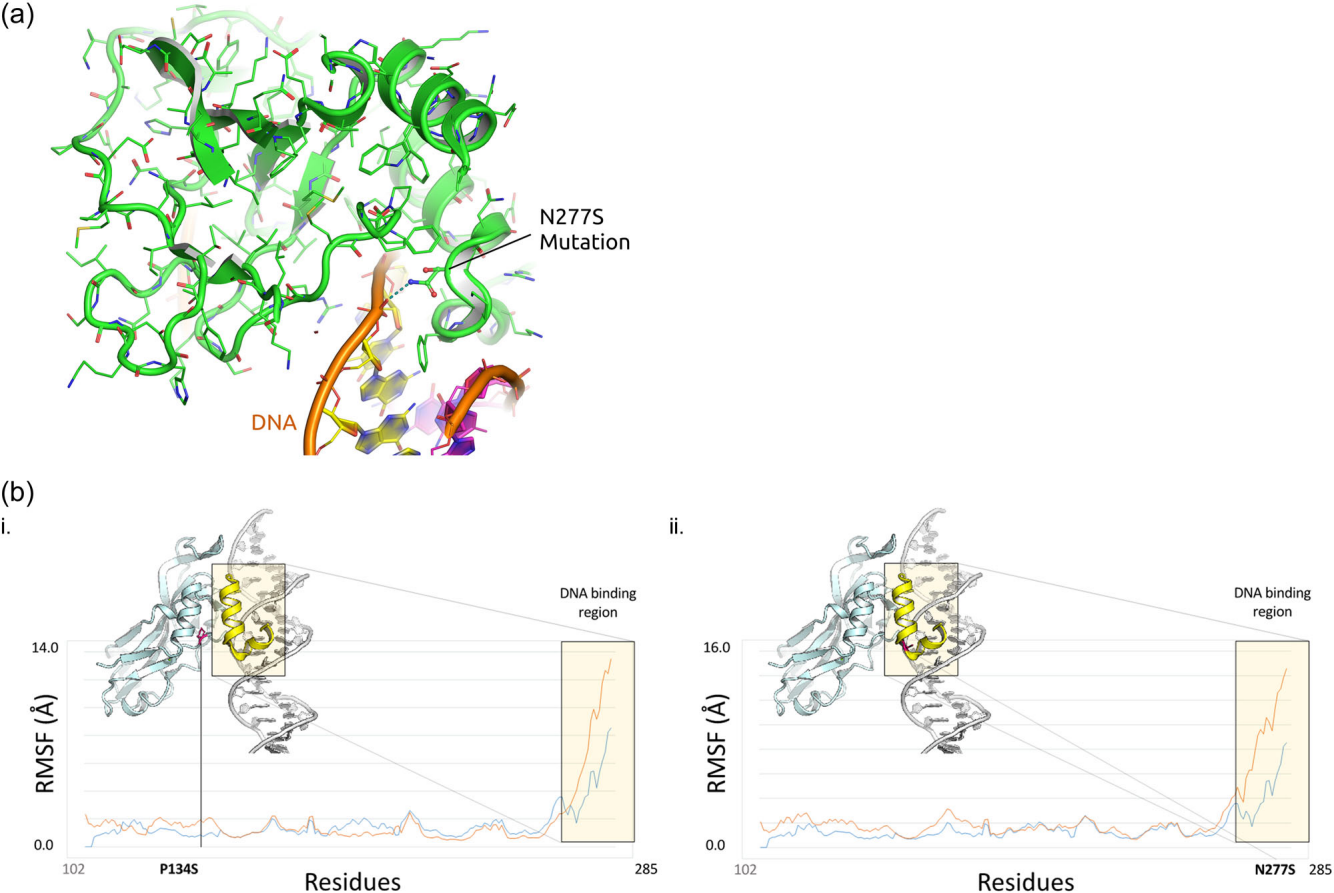
Molecular modeling of *TBX3* missense variants. (a) Structural effect of p.N277S mutation. Asparagine 277 is responsible for a key hydrogen‐bond interaction between the C‐terminal domain helix of TBX3 and DNA. The p.N277S mutant is not able to engage the same interaction. (b) Movements and flexibility of the C‐terminal DNA binding region of (i.) WT and p.P134S (ii.) WT and p.N277S mutants of TBX3. The RMSF Plot shows a significant increase of flexibility of TBX3 upon p.P134S and p.N277S mutations.

To get additional mechanistic insights on the biological effects of the expression of TBX3 variants, we carried out a molecular modeling of the corresponding protein structures. Three‐dimensional (3D) models of TBX3 p.N277S and p.P134S were built using the homology modeling approach implemented in the PyMod 3 package, starting from the crystal structure of human TBX3 bound to a palindromic DNA site (PDB: 1H6F). The model for p.N277S (Figure 5a) showed that the change of Asparagine 277 into Serine is responsible for the loss of an important hydrogen‐bond that stabilizes the interaction between the C‐terminal domain helix of TBX3 and DNA. The loss of this interaction, in turn, is supposed to destabilize the DNA‐binding properties of the TXB3 transcription factor. Apparently, the p.P134S mutation seems to easily accommodate serine within the pocket of TBX3 without the lack of disruption in intramolecular or DNA contacts. However, given the unique characteristics of Proline in the context of protein structures, and since the P134 and N277 residues are both located at the interface between the N‐terminal and C‐terminal subdomains of TBX3 (Figure 5b), we hypothesized that both mutations affected the dynamic behavior of TBX3. Thus, we performed comparative WT versus Mutant 1000 ns Molecular Dynamics simulations to assess the movements and flexibility of the C‐terminal DNA binding region of TBX3 in both p.P134S and p.N277S variants. The results showed that the both alterations are responsible for a significant increase of flexibility of the C‐terminal DNA binding domain, which in turn provides the evidence of a destabilization effect on DNA binding (Figure 5b).

Since the p.T539M and p.E695R variants are in a disordered region, it was not possible to rationalize their effect from a structure/function relationship perspective.

Collectively, the characterization of developmental defects along with the IF results obtained in our humanized flies, indicate that the severity of the single phenotypes and lethality associated with the expression of either TBX3^WT^, TBX3^T539M^ and TBX3^E695R^ could depend on a persistent nuclear localization of these proteins on chromatin. On the contrary, the less efficient localization of both TBX3^P134S^ and TBX3^N277S^ variants that, consistently with the modeling prediction, bind to the chromatin weakly, renders these mutant TBX3 proteins be less toxic. Accordingly, flies expressing these latter variants are mostly viable and elicit a milder phenotype compared to flies expressing the other variants. These data thus define the inverse functional correlation described here in which the overexpression of a TBX3 protein that retains a wild‐type function in human cells (TBX3^WT^; TBX3^T539M^ and TBX3^E695R^) causes a severe phenotype in Drosophila, while the overexpression of a TBX3 variant which is dysfunctional in human cells (TBX3^P134S^ and TBX3^N277S^) results only into a mild phenotype in Drosophila.

## 3 DISCUSSION

Herein, we provide clinical, genomic, bioinformatics and functional evidence that the novel *TBX3*:c.400 C > T (p.P134S) causes UMS. To our knowledge, this is the first description of UMS in an antenatal setting. Although the mother displayed the typical, though mild UMS features, she remained misdiagnosed until molecular studies after the disclosure of the fetal anomalies. Interestingly, also the proband's father, who was likely affected by the same disorder, was never recognized and died in late adulthood for an unrelated disease. This family prompts to hypothesize that UMS is more common than expected. Indeed, even if the syndrome was firstly described in the '70 s (Gonzalez et al., 1976; Pallister et al., 1976) and associated with *TBX3* alterations in 1996 (Bamshad et al., 1996), to date only about 130 affected cases have been reported in the literature (Zhang et al., 2023). As testified in this work, it is possible that UMS is easily misdiagnosed both in the prenatal and postnatal setting due to its marked clinical variability. Patients with a mild phenotype have been reported presenting with deformities in the distal segments of the little fingers of both hands, but with no other manifestations (Zhang et al., 2023). Indeed, it is only after the postpubertal age that the other typical signs (e.g., breast hypoplasia, scarce axillary hair, decreased perspiration) can be more properly recognized. As a result, various affected individuals could be present in a family and remain unrecognized until one relative exhibits high‐expressive symptoms, even prenatally. Accordingly, we suggest to carefully search for genitalia, breast, nipple and other skin adnexa sign in all cases of apparently isolated post‐axial limb anomalies also in the prepubertal cases.

The *TBX3* constitutional mutational spectrum resulted to be mainly composed of truncating, missense and splicing variants. While truncating *TBX3* point alterations map along the entire coding sequence, the cluster of pathogenic/likely pathogenic missense variants maps in the T‐Box domain (Figure 2). On the other hand, the cluster of benign/likely benign missense changes is located downstream the T‐box. In line with this observation, our molecular findings on *TBX3* humanized flies help in understanding as to why missense changes in the T‐box domain are expected to be UMS causative compared to those located outside the T‐box. Indeed, among the selected UMS‐causing *TBX3* missense variants, those mapping in the T‐box (i.e., p.P134S and p.N277S) were predicted to affect the dynamic behavior of TBX3 and its binding to DNA. The resulting impairment of movements and flexibility of the C‐terminal DNA binding region of TBX3 could be then responsible for most severe defects associated with UMS. Conversely, our findings on the missense changes located downstream the T‐box (i.e., p.T539M and p.E695R) show that they functionally mimic the expression of the wild type *TBX3*, thus confirming that an intact T‐box domain is required for the role of *TBX3* in proper limb development. Interestingly, all humanized viable flies expressing a normal TBX3 protein or TBX3 mutant variants in a wild‐type genotype elicited a wing phenotype similar to loss of function mutants of the fly ortholog *bifid/omb* (Sen et al., 2010), indicating that the human *TBX3* genes are sensitive to dose dependent effects and abolish the function and/or localization of the endogenous OMB protein (see also Supplementary Figure 5). This is not unexpected as the expression of variants of the TBX3 closest paralog, TBX2, were also demonstrated to behave as hypomorphic alleles when tested in cultured cells and in vivo in flies, thus suggesting that they acted in a loss‐of function manner (Liu et al., 2018).

Despite the effects of P134S and N277S depend on their loss of function mechanism that destabilize the DNA‐binding properties of the TXB3, our cytological observations indicate that these variants affect the expression of the protein in *Drosophila* tissues in a distinguishable manner. The wild‐type TBX3 shows a robust, nuclear/chromatin localization, which is consistent with its well‐described role as a transcription factor (Sowden et al., 2001). As expected, this localization is weakened by the p.P134S variant in the R2 domain that it is also in line with our *in silico* molecular modeling. The reason as to why the p.N277S variant forms aggregates is still unclear. We can hypothesize that the loss of a key hydrogen bond that normally guarantees the interaction between the C‐terminal domain helix of TBX3 and DNA predicted by the molecular modeling, could restrain the TBX3 ability to bind diffusely on chromatin leading to the formation of foci in which TBX3 accumulates. We can envisage that these foci resemble nuclear speckles that are normally involved in the regulation of various steps of gene expression and/or splicing in the nucleus and localize at specific and coordinately expressed genes to favor transcription regulation interaction with splicing factors (Hirose et al., 2023). Indeed, the observation that TBX3 regulates splicing in vivo gives further support to our latter hypothesis (Kumar et al., 2014). Regardless the different effects on TBX3 localization, both p.P134S and p.N277S variants influence the ability of TBX3 to efficiently bind to chromatin thus explaining the loss of function nature of these variants.

Our finding that the expression of the N277S‐encoding transgene induces phenotypes that are milder than those elicited by P134S expressing flies (see Table 1), suggests that among the selected alteration, the p.N277S variant is the strongest *TBX3* allele. This conclusion could suggest that the p.N227S variant might be nonviable in humans. In line with this hypothesis, it has never been found at constitutional level. Moreover, the dramatic effects of its localization (as shown in fly tissues) could also hinder a proliferative transcriptional program of different cell lineages thus explaining its association with tumor. Finally, the finding on the p.T539M, that maps downstream the T box domain (and therefore likely non pathogenic for UMS) and outside the R1 domain, is found only in tumors might suggest that a potential association of this variant with tumor development is not dependent on an impaired function as a transcription factor of TBX3.

Taken together, our results enhance our understanding of the molecular basis and the clinical spectrum of UMS and contribute with the first humanized *Drosophila* model for *TBX3* as an instrument for the comprehension of molecular mechanisms that underlie the disease.

## 4 MATERIALS AND METHODS

### 4.1 Genetics analyses

During genetic counselling, the woman, her husband and mother gave informed consent for the genetic analyses, which was approved by local ethic committees in accordance with the principles of the Declaration of Helsinki. Genomic DNA from both peripheral blood and from abortion material (i.e., a fragment of fetal skin) was extracted by standard methods. ES was performed on the parents' DNA by Nextera DNA Exome with Enrichment (Illumina, San Diego, USA) and then run on NextSeq. 2000 (Illumina). Sequencing reads were aligned to the human reference genome (UCSC hg19) by BWA (v0.7.7‐isis‐1.0.2) (Illumina). Variant calling was performed by GATK Variant Caller (v1.6‐23‐gf0210b3). The DNA variants were annotated by eVai v2.5 (enGenome, Pavia, Italy). DNA changes were filtered by MAF < 0.01 (GnomAD v2.1) and classified according to ACMG‐AMP criteria(Richards et al., 2015). Sanger sequencing was employed for validating the filtered variants and for testing them on the fetal DNA, by the use of the following PCR primers' pair:

TBX3_Ex2_FW: 5′‐AGTTGCCTCACTCTGAAACA‐3′

TBX3_Ex2_Rv: 5′‐ AGAAGCAAAGGAGAAGTCTGG‐3′

### 4.2 *Drosophila* transgenic flies generation and crosses

TBX3‐WT, ‐P134S, ‐N277S, ‐T539M and E695R encoding cDNAs were generated by standard gene synthesis at Genewiz‐Azenta Life Science (Leipiz, Germany). Gene synthesis included added 5' (XhoI) and 3' (XbaI) sequences (see Supplementary Material 2 for full sequences). After sequence verification, cDNAs were cloned into pUAS‐attB vector via 5' XhoI and 3' XbaI and delivered as a mini‐scale DNA sample. Constructs were injected via PhiC31 integrase‐mediated transgenesis system into P[attP.w[+]. attP]JB37B embryos that harbor the phage attachment site (attP) on the second chromosome (at the 37B7 cytogenetic site). Transgenic flies were selected according to the mini‐w+ marker present on the transgenic construct. At least four different lines were established for each injection. Ectopic expression of either TBX3‐WT, ‐P134S, ‐N277S, ‐T539M or E695R variants was obtained by crossing UAS‐TBX3‐WT and UAS‐TBX3 variants to *eyeless*‐*GAL4* and GMR‐*GAL4* (for phenotype analysis of eyes), *engrailed‐GAL4* and MS1096‐*GAL4* (wing phenotype) and *Tubulin*‐*GAL4* (larval brain phenotype) driver lines, all obtained from the Bloomington Stock Center. Information on these lines is available at Flybase (http://flybase.bio.indiana.edu/). Stocks were maintained and crosses were made on standard *Drosophila* medium at 18°, 25°, 29°C.

### 4.3 *Drosophila* chromosome cytology and immunostaining

To obtain larval neuroblast preparations for immunostaining, brains were dissected in 0.7% sodium chloride then fixed for 10 min with 3.7% formaldehyde, 45% acetic acid and squashed in the same fixative. Slides were frozen in liquid nitrogen and, after flipping off the coverslip, were immersed in cold EtOH, washed in TBS‐Triton 0.1% twice for 5 min and incubated overnight at 4°C with the appropriate antibodies. To obtain polytene chromosomes for immunostaining, salivary glands were dissected in 0.7% sodium chloride then fixed for 2 min with 1.7% formaldehyde, 45% acetic acid and squashed in the same fixative. Slides were frozen in liquid nitrogen and, after flipping off the coverslip, were immersed in cold TBS, washed in TBS‐Triton 0.1% twice for 5 min and incubated overnight at 4°C with appropriate antibodies.

For antibody immunostaining, brain, wing discs and polytene squashes were incubated with rabbit anti‐TBX3 (Invitrogen, 1:100) and anti‐OMB (1:100), kindly provided by Prof. Pflugfelder (Mainz University). Slides were then washed twice in TBS‐Tween 0.1% for 15 min and incubated for 2 h at room temperature with anti‐rabbit (Invitrogen, 1:300). For anti‐OMB immunostaining slides were treated with the Antigen Retrieval Buffer (Leica) (Cipressa et al., 2016). All slides were then mounted in Vectashield medium H‐1200 with DAPI to stain DNA. Chromosome preparations were analyzed using a Zeiss Axioplan epifluorescence microscope (CarlZeiss, Oberkochen, Germany), equipped with a cooled CCD camera (Photometrics, Woburn, MA). Gray‐scale digital images were collected separately, converted to Photoshop format, pseudocolored, and merged. To quantify the nuclear fluorescence intensity after anti‐TBX3 immunostaining, we used the ImageJ software (National Institute of Mental Health, Bethesda, Maryland, USA) and measured the fluorescence of selected areas and the fluorescence of a close nuclei‐free region to correct for background fluorescence. For each genotype, the quantification was carried out on at least 120 nuclei.

### 4.4 Protein extracts and Western blot analysis

Protein extracts from *Drosophila* larval brains and adult eyes were obtained by dissecting 10 larval brains and/or 10 adult heads in 0.7% NaCl and homogenizing them in 20 μl of RIPA buffer. Protein samples were loaded into a 4‐20% Mini‐PROTEAN TGX precast gel to perform electrophoresis (SDS‐PAGE) and blotted using the Trans‐Blot® TurboTM Transfer System on a nitrocellulose membrane (Thermo Scientific). Filters were blocked in 5% nonfat dry milk dissolved in 0.1% Tween‐20/PBS for 30 min at RT and, then, incubated with anti‐Giotto (1:5000; Rabbit; (Giansanti et al., 2006)) and anti‐TBX3 (Invitrogen, 1:1000) overnight at 4°C. The membranes were then incubated with HRP‐conjugated anti‐Mouse (Jackson immuno‐reasearch 1:5000) and anti‐Rabbit (Bethyl laboratories, 1:5000) for 1 h at RT and then washed again 3 times with 0.1%Tween‐20/PBS. The chemiluminescent signal was revealed through either SuperSignalTM West Femto or SuperSignalTM West Pico substrate (Thermo ScientificTM) using the ChemiDoc scanning system (Bio‐Rad). Band intensities were quantified by densitometric analysis using the Image Lab 4.0.1 software (Bio‐Rad). WB was repeated independently at least three times.

### 4.5 Total RNA extraction, cDNA amplification and qPCR

*Drosophila* RNA was isolated from brain of third instar larvae. Brains (10 brains/genotype) were dissected in triplicate and RNA extracted using TRIzol (TRI Reagent® SIGMA Life Science, Sigma‐Aldrich). To validate the expression levels of *TBX3* transcripts, equal amounts of cDNA were synthesized from 300 ng of total RNA for each sample by using the iScript™ cDNA Synthesis Kit (Bio‐Rad, Hercules, CA, USA). Thirty nanograms of cDNA per reaction were analyzed for semi‐qPCR using the SsoAdvanced™ Universal SYBR® Green Supermix Kit (Bio‐Rad) following the manufacturer's protocol. The thermal cycling conditions were: 50°C (2 min), 95°C (10 min) followed by 40 cycles at 95°C (15 s), 60°C (1 min), and 95°C (15 s), 60°C (1 min) 95°C (15 s), and 60°C (15 s). The specificity of the reaction was verified by melting curve analysis. The PCR primers used were:

TBX3_FW 5′‐GCAGCTTTCAACTGCTTCG‐3′

TBX3_RV 5′‐CCTCGCTGGGACATAAATCT‐3′

rp49 was amplified as a reference transcript using the following primers:

rp49_FW 5′‐CCGCTTCAAGGGACAGTATCT‐3′

rp49_RV 5′‐ATCTCGCCGCAGTAAACGC‐3′

PCR reactions were carried out in the ABI Prism 7300 System (Applied Biosystems, Foster City, CA, USA). Data processing was performed using the ABI SDS v2.1 software (Applied Biosystems). The critical threshold value was noted for each transcript and normalized to the internal control. The fold change was calculated using the comparative 2(^−ΔΔCt^) method (Livak & Schmittgen, 2001).

### 4.6 *Drosophila* eye and wing analysis

Adult wings were dissected in isopropanol and mounted on microscope slides with mounting Canada balsam (Sigma‐Aldrich). Adult eyes and wings images were taken with a Zeiss Stemi 508 stereo microscope equipped with an Axiocam camera and acquisition Zen program. Image processing and eye dimension measurements were performed with ImageJ through manually eye area selection and surface measurement.

### 4.7 Statistics and reproducibility

Data were presented using the mean ± standard error (SE) obtained from at least three independent experiments. All the statistical analyses were performed with Sigmaplot 11.0 (Systat Software, Imc., Chicago, IL, USA). The comparison between the two groups was analyzed by the Student t‐test. The results were considered statistically significant when the p values were <0.05.

### 4.8 Molecular modeling

Molecular modeling simulations of the molecular models of wild‐type, p.P134S and p.N277S mutants (mutated with PyMod (Janson & Paiardini, 2021)), derived from the crystal structure of human TBX3 (PDB: 1H6F; Ref), downloaded from Brookhaven Protein Data Bank (PDB) were performed. The structures were energy minimized in vacuum, using the Amber force field and Gromacs package (Van Der Spoel et al., 2005). Briefly, after an initial minimization performed to allow added hydrogens to adjust to the crystallographically defined environment, a 5,000‐step steepest descent minimization without periodic boundary conditions was performed, until the maximum derivative was less than 0.01 kJ Mol−1 Å−1. Then, the system was soaked by a water box of 97 × 97 × 97 Å3 dimensions, filled with a total of 20,000 water molecules and counterions added to neutralize the net negative charge of the protein and obtain a concentration of 0.01 M Naþ∕Cl − . Energy minimization was then performed for 5,000‐step steepest descent, but in this case periodic boundary conditions were introduced. With the greatest strain dissipated from the system, the next step was to let the solvent adapt to the protein, while keeping the non‐hydrogen atoms of the proteins fixed to the reference positions. For this purpose, a position restrained molecular modeling was performed for 10 ns at 200 K. After pressure coupling, the system was gradually heated during 500 ps. Finally, a production simulation was carried out at 300 K for 1000 ns, with a time step for integration of 0.2 fs. Bond lengths were constrained by using the LINCS algorithm. Standard quality assurance tests were carried out by means of the Gromacs package for the convergence of the following thermodynamic parameters: temperature, potential, and kinetic energy. Convergence was also checked in terms of the structure, through the rmsd against the starting structure. Next to that, it was checked that during molecular modeling simulation there were not interactions between adjacent periodic images, because such interactions could lead to unphysical effects. Molecular modeling parameters files and trajectory files are available upon request to the authors.

## HUMAN RIGHTS AND ANIMAL RESEARCH

This study was conducted according to the ethical principles for medical research involving human subjects according to the Declaration of Helsinki.

## AUTHOR CONTRIBUTIONS

Irene Bottillo interpreted the data and wrote the main manuscript; Andrea D'Alessandro acquired the data; Maria Pia Ciccone acquired the data; Gianluca Di Giacomo and Evelina Silvestri interpreted the data; Marco Castori and Francesco Brancati interpreted the data and critically revised the manuscript; Gianluca Cestra acquired the data, interpreted the data and revised the manuscript; Silvia Majore acquired the data, interpreted the data and critically revised the manuscript; Alessandro Paiardini acquired the modeling data; Andrea Lenzi established the partnership and revised the manuscript; Giovanni Cenci interpreted the data, supervised the study and wrote the main manuscript; Paola Grammatico obtained funding and supervised the study.

## FUNDING

This work has been supported by grants from Institute Pasteur of Rome and Italy Ministry of University and Research (PRIN, N 202227SYBW) to Giovanni Cenci, an European Union–NextGenerationEU grant through the Italian Ministry of University and Research (PNRR‐M4C2‐I1.3 Project PE_00000019 “HEAL ITALIA” to Paola Grammatico CUP B53C22004000006), a grant from Sapienza University of Rome (project N. 000055_23_RS_ BOTTILLO_Ateneo_ ProgPiccoli 2022) to Irene Bottillo; CNR (DBA. AD005.225‐NUTRAGE‐FOE2021), CNR (Flagship Project Interomics) and EU funding within the MUR PNRR “National Center for Gene Therapy and Drugs based on RNA Technology” (#CN00000041 CN3 RNA) to Gianluca Cestra; Progetti Ateneo Sapienza University of Rome (RM1221815D52AB32) and Italy Ministry of University and Research PRIN (CUP: 2022N3JXLA) to Alessandro Paiardini.

## CONFLICT OF INTEREST STATEMENT

Conflicts of interest have been disclosed by any of the authors.

## Supporting information

Supporting information.

Supporting information.

Supporting information.
