# Supplementary material for: An inherited TBX3 alteration in a prenatal case of ulnar‐mammary syndrome: Clinical assessment and functional characterization in Drosophila melanogaster
Source: J Cell Physiol. 2024 Sep 25;239(12):e31440. doi: 10.1002/jcp.31440 (PMC11649972; doi:10.1002/jcp.31440)
Supplement: Supplementary file 1 — Supporting information. [file JCP-239-0-s003.docx]

**An inherited TBX alteration in a prenatal case of ulnar-mammary syndrome: clinical assessment and functional characterization in Drosophila melanogaster**

Bottillo Irene, D’Alessandro Andrea^,^, Ciccone Maria Pia, Cestra Gianluca^,,^, Di Giacomo Gianluca, Silvestri Evelina, Castori Marco, Brancati Francesco^,^, Lenzi Andrea, Paiardini Alessandro, Majore Silvia, Cenci Giovanni^,#^, Grammatico Paola

**Supplementary Figures**


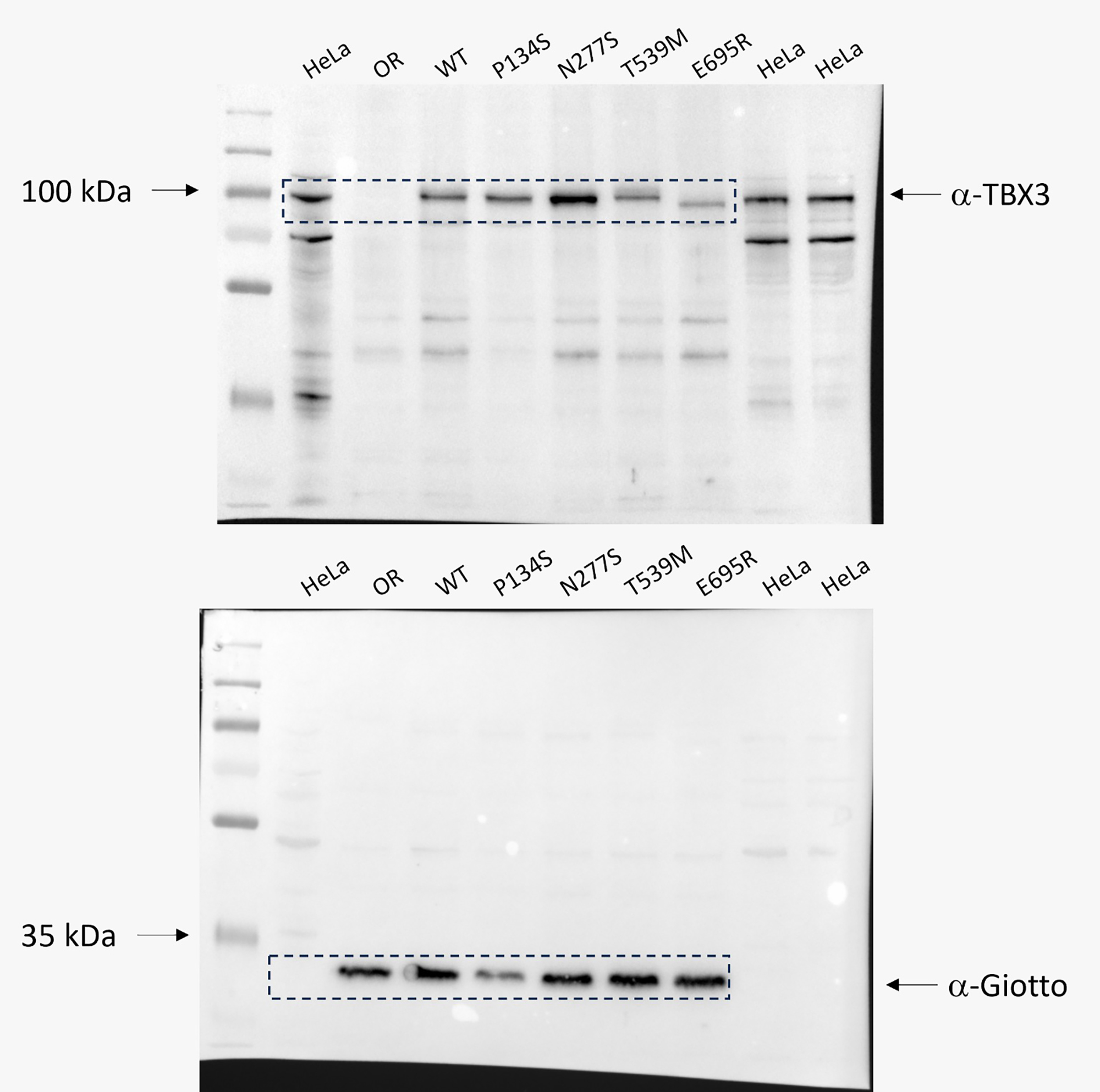


**Supplementary Figure 1**. **Uncropped blot images for Figure 3B**. Rectangles indicate selected areas used for panel B


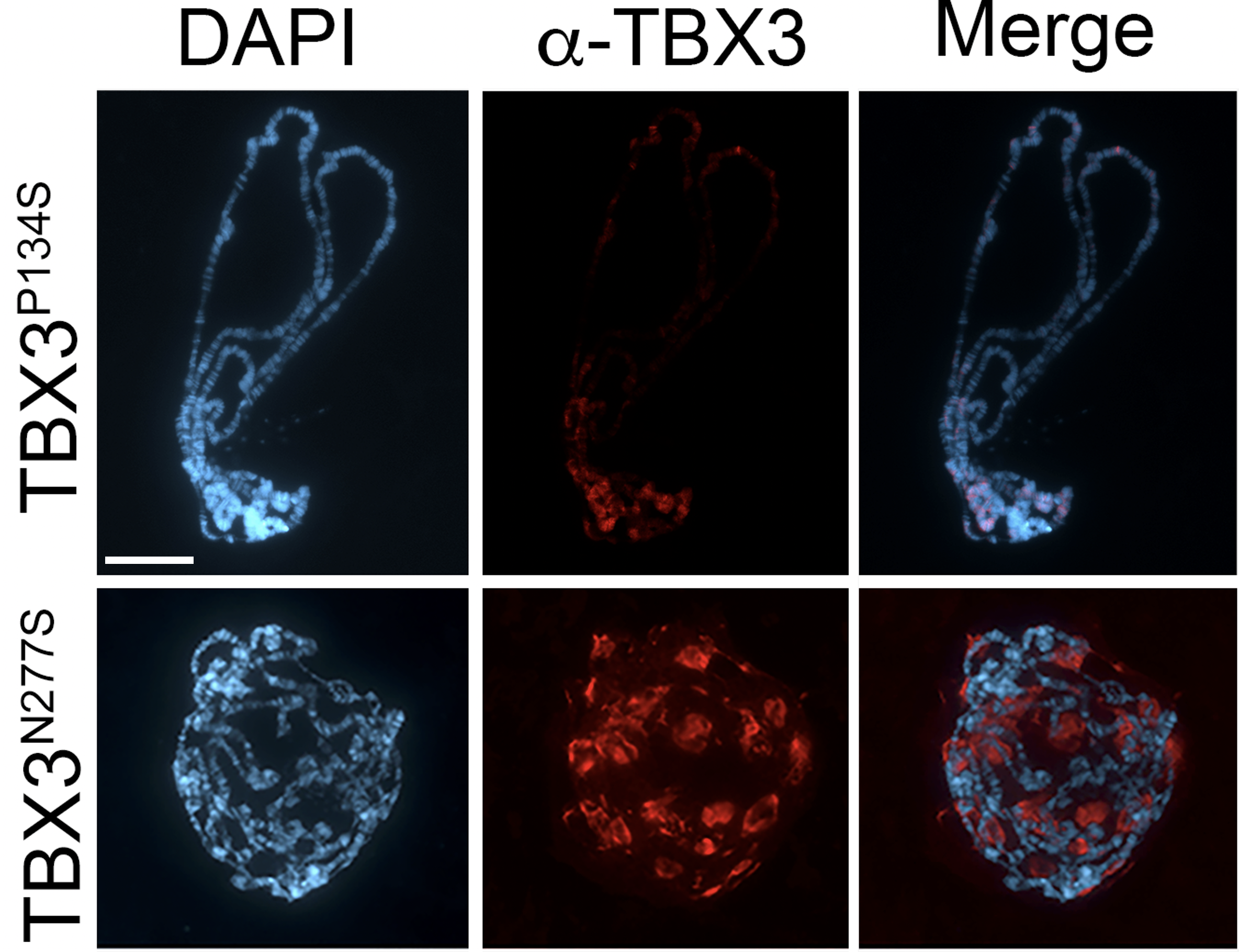


**Supplementary Figure 2**. **Localization of mutant TBX^P134S^ and TBX^N277S^ proteins in larval polytene nuclei.** *Eyeless GAL>UAS-TBX* ***^P134S^*** and -*TBX^N277S^* expressing polytene nuclei stained with anti-TBX (red) and DAPI (blue; for DNA). Note that, in line with the localization on neuroblasts (Figure 4), while TBX^P134S^ is associated with polytene chromosome arms, TBX^N277S^ forms distinct aggregates that partially localize on polytene chromosomes (see merge). Scale bar: 25μM


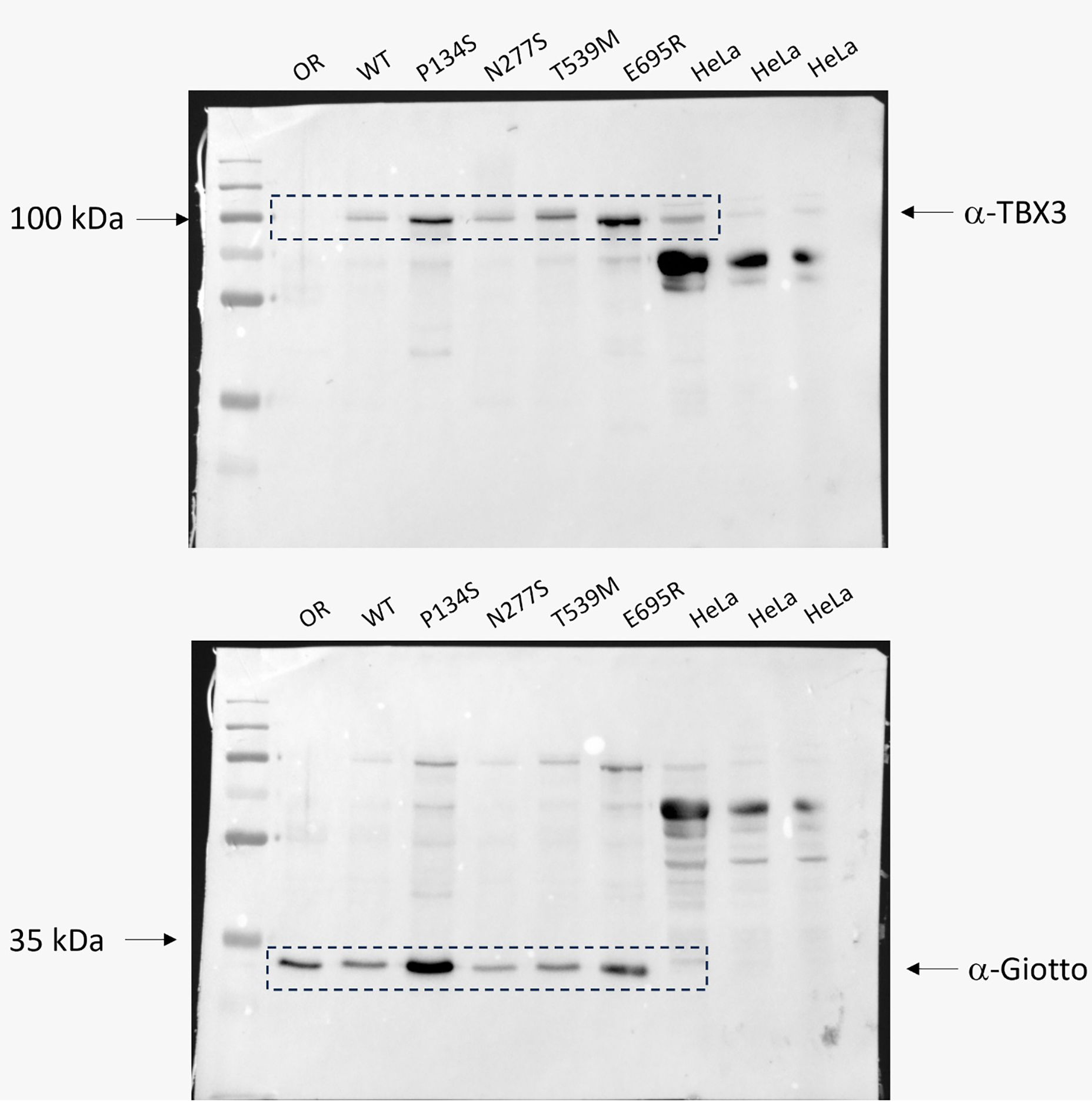


**Supplementary Figure 3. Uncropped blot images for Figure 4 B**. Rectangles indicate the selected areas used for panel C


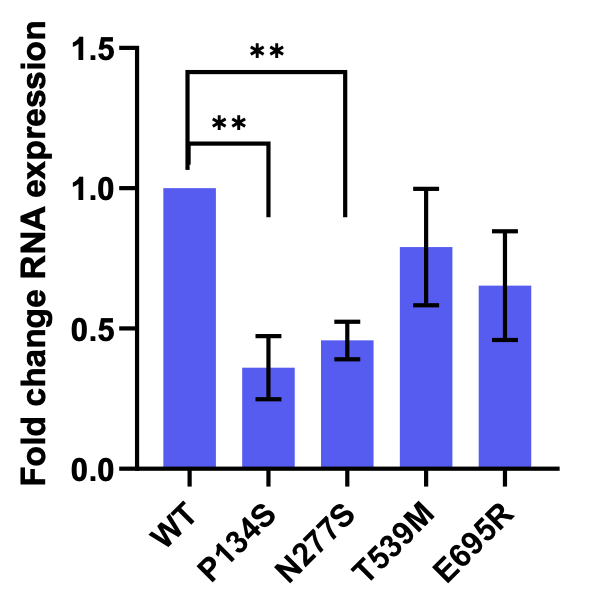


**Supplementary Figure 4. mRNA expression analysis of the different transgenes**. qPCR of RNAs extracted from humanized larval brains. Note that N277S encoding transgene shows a ~ 2 fold reduction of transcription, despite its protein levels are more abundant than the other variants. See text for further details. ** p<0.01


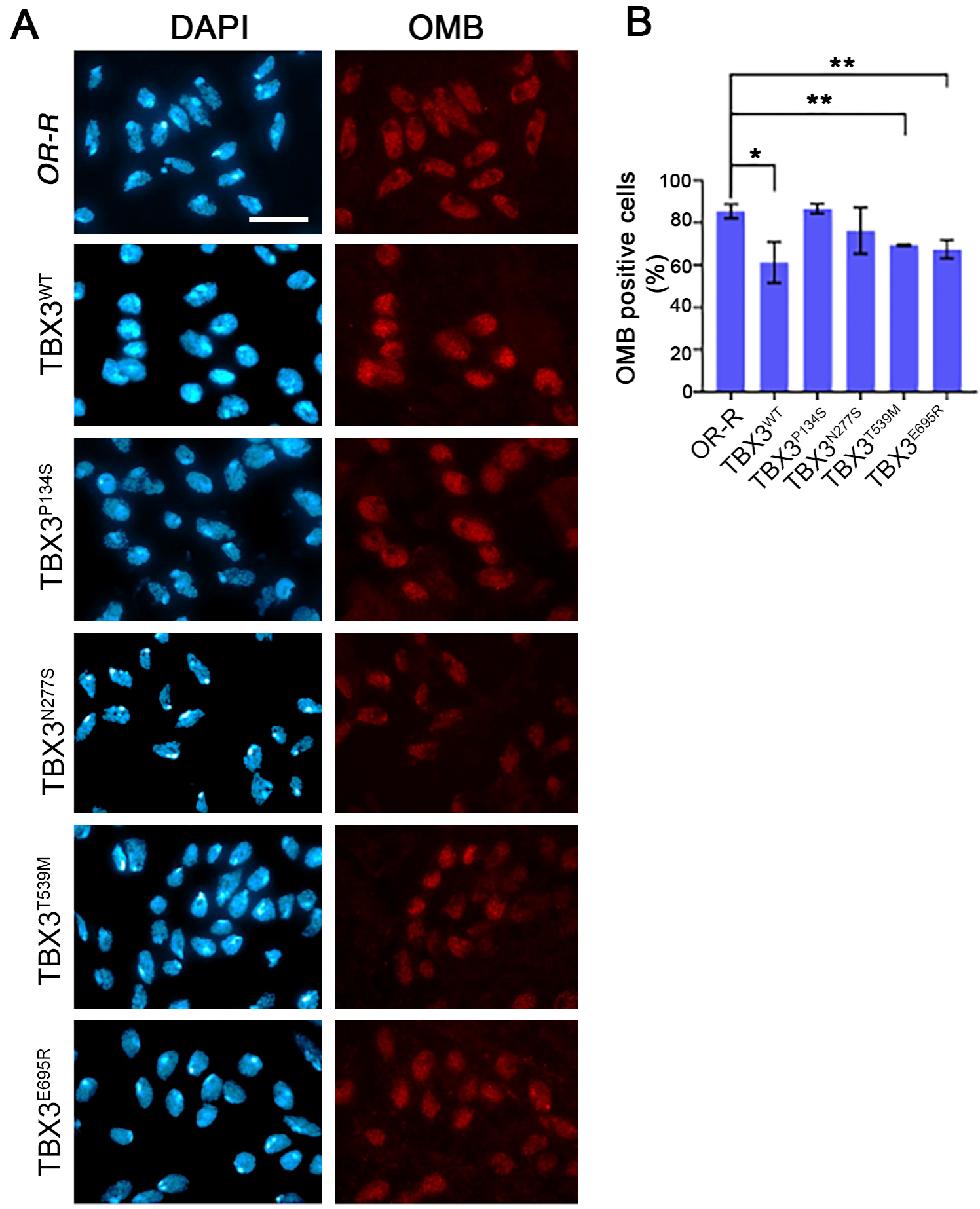


**Supplementary Figure 5. Analysis of OMB expression in cells from the different humanized flies**. **A**. Immunolocalization of OMB on humanized lines. OR-R is the wild-type control. Scale Bar: 10μm. **B**. Quantification of cells immunostained for OMB. Note that that while OMB localizes on the nucleus in the ~80% of control disc cells (n=700), it decorates ~60% of TBX3-WT, TBX3-T539M and TBX3-E695R wing disc nuclei. In contrast, OMB localization in TBX3-P134S (~85% of nuclei) and in TBX3-N277S (~75% of nuclei) was almost indistinguishable from control (n=~700). This suggests the overexpression of either WT or mutant TBX3 variants that are functionally equivalent to WT, suppresses the OMB localization, as also confirmed by the *omb* mutant*-*like phenotype associated with the overexpression of these human transgenes in flies. Interestingly, the OMB localization was only slightly affected in TBX3-P134S and TBX3-N277S expressing flies with respect to the other variants, in line with the observations that the expression of these variants induces a less sever phenotype. Error bars represent the standard errors of the mean. (* p<0.05,, **p<0.01 Student t-test); Bar =
