# Supplementary material for: An inherited TBX3 alteration in a prenatal case of ulnar‐mammary syndrome: Clinical assessment and functional characterization in Drosophila melanogaster
Source: J Cell Physiol. 2024 Sep 25;239(12):e31440. doi: 10.1002/jcp.31440 (PMC11649972; doi:10.1002/jcp.31440)
Supplement: Supplementary file 2 — Supporting information. [file JCP-239-0-s001.pdf]

TBX3 variant NM_005996.4					Patients			Clinical features																				
								Obesity	Eyes	Teeth	Heart	Chest	Breast		Abdomen	Gastrointestinal	Genitourinary	Upper limbs				Feet	Apocrine gland hypoplasia	Perspiration	Axillary Hair	Nails	Neurologic features	Endocrine features
Exon	Nucleotide change	Protein change		Reference	N°	Sex	Age	Obesity	Scant lateral eyebrows/epicanthic folds/hypertelorism/blue sclerea	Teeth defects/ high arched palate	Heart defect	Hypoplastic scapula/ clavicle/ pectus carenatum/scapula alata	Breast/nipple hypoplasia (females)	Nipple/areola hypoplasia (both sexes)	Inguinal hernia	Anal atresia/stenosis	Genitalia anomalies	Ulna absence/deformity	Radius bowing	Humerus shortening	Digitis' anomalies	Short fourth and fifth toes	Axillary apocrine gland hypoplasia	Diminished perspiration	Axillary Hair absence	Nails' anomalies	Pituitary hypoplasia	Delayed puberty
7	c.1920_1921dup	p.Pro641ArgfsTer229	longer protein	M.M. Al-Qattan et al,2020	pt	F	10y	-	-	-	-	-	-	-	-	-	-	-	-	-	+	-	-	-	-	-	-	-
7	c.1857delC	p.Tyr620ThrfsTer249	longer protein	Bamshad M et al,1999	1	M		-	-	-	-	-	-	-	-	-	-	-	-	-	+	-	-	-	-	-	-	-
					2	F		-	-	-	-	-	+	+	-	-	-	-	-	-	+	-	-	+	+	-	-	-
					3	F		-	-	-	-	-	+	+	-	-	-	-	-	-	+	-	-	+	+	-	-	-
					4	F		-	-	-	-	-	+	+	-	-	-	-	-	-	-	-	-	+	+	-	-	-
					5	F		-	-	-	-	-	-	-	-	-	-	-	-	-	+	-	-	-	-	-	-	-
					6	F		-	-	-	-	-	+	+	-	-	-	-	-	-	-	-	-	+	+	-	-	-
					7	M		-	-	-	-	-	-	-	-	-	+	-	-	-	+	-	-	+	+	-	-	-
					8	F		-	-	-	-	-	+	+	-	-	-	-	-	-	+	-	-	+	+	-	-	-
					9	F		-	-	-	-	-	+	+	-	-	-	-	-	-	-	-	-	+	+	-	-	-
					10	F		-	-	-	-	-	+	+	-	-	-	-	-	-	-	-	-	+	+	-	-	-
					11	F		-	-	+	-	-	+	+	-	-	-	-	-	-	+	-	-	+	+	-	-	+
					12	M		-	-	-	-	-		+	-	+	-	+ (left)	-	-	+	-	-	+	+	-	-	+
6	c.1710+2T>A	-	splicing effect	Bamshad M et al,1999	1	M		-	-	-	-	-		+	-	+	+	-	-	-	+	-	-	+	+	-	-	+
					2	M		+	-	-	-	-		+	-	+	+	-	-	-	+	-	-	+	+	-	-	+
					3	M		-	-	-	-	-		+	-	+	+	-	-	-	+	-	-	+	+	-	-	+
					4	M		+	-	-	-	-		+	+	-	+	-	-	-	+	-	-	+	+	-	-	+
6	c.1423C>T	p.Gln475Ter	truncating	George A. Tanteles et al,2017	father	M		-	-	-	+	-	-	n	-	-	-	-	-	-	+	-	-	-	-	-	-	+
					Twin 1	M	14y	-	+	-	-	-	-	-	-	-	+	-	-	-	+	-	-	-	-	-	-	+
					Twin 2	M	14y	-	+	-	-	-		+	-	-	+	-	-	-	+	-	-	-	-	-	-	+
6	c.1294_1301dup	p.Pro437AlafsTer178	truncating	Bamshad M et al,1999	1	M		-	-	-	-	-	-	n	-	-	-	-	-	-	+	-	+	+	+	-	-	-
					2	M		-	-	-	-	-	-	-	-	-	-	-	-	-	+	+	+	+	+	-	-	+
					3	F		-	-	-	-	-	+	-	-	-	-	-	-	-	+	-	+	+	+	-	-	-
					4	F		-	-	-	-	-	-	-	-	-	-	+	-	-	+	-	+	+	+	-	-	+
6	c.1190del	p.Phe397SerfsTer215	truncating	ClinVar	pt	n		-	-	-	+	-	-	-	-	-	-	-	-	-	-	-	-	-	-	-	+	-
6	c.1136dup	p.Ser380ValfsTer7	truncating	Meneghini V et al.,2006	A	M		-	-	-	+	-	-	-	-	-	+	-	-	-	+	-	-	-	-	-	-	-
					B	M		-	-	-	+	-		+	-	-	+	+	-	-	+	-	-	-	+	+	-	-
					C	F		-	-	+	-	-	+	+	-	+	-	-	-	-	+	-	-	+	+	-	-	-
6	c.1070_1080del	p.Ala357GlufsTer26	truncating	ClinVar				n	n	n	n	n	n	n	n	n	n	n	n	n	n	n	n	n	n	n	n	n
6	c.1061_1064del	p.Glu354Alafs*257	truncating	Zhang_2023	proband	M	13y	-	-	-	+	-		-	-	-	+	+	-	-	+	-	-	+	n	-	+	-
					mother	F		-	-	-	n	-	-	-	-	-	-	-	-	-	+	-	-	-	n	-	-	-
5	c.1039+1G>A	-	splicing	ClinVar				n	n	n	n	n	n	n	n	n	n	n	n	n	n	n	n	n	n	n	n	n
5	c.992dupA	p.Ala332GlyfsTer23	truncating	Shelagh Joss et al. 2011	proband	F	7m	-	-	-	-	-	-	n	-	-	-	+ (right)	-	-	+	-	-	-	-	-	-	-
					mother	F	24y	-	-	+	-	-	+	+	-	-	-	-	-	-	+	-	-	-	-	-	-	-
					grandmother	F	59y	-	+	-	-	-	-	+	-	-	-	-	-	-	-	-	-	-	+	-	-	-
5	c.1028C>A	p.S343Ter	truncating	Bamshad M et al,1999	1	F		-	-	-	-	-	+	-	-	+	-	-	-	-	+	-	-	+	+	-	-	-
					2	M		-	-	-	-	-	-	-	-	+	+	-	-	-	+	-	-	+	+	-	-	-
5	c.991C>T	p.Gln331Ter	truncating	Shelagh Joss et al,2011	proband	M	4y	-	-	-	+	-		+	-	-	+	+ (bilateral)	-	-	+	-	-	-	-	-	+	+
					sister	F	11y	-	-	-	-	-	+	+	-	-	-	-	-	-	-	-	-	-	-	-	-	+
					mother	F	39y	-	+	-	-	-	-	-	-	-	-	-	-	-	-	-	-	+	+	-	-	-
4	c.857_858delCT	p.Thr286ArgfsTer20	truncating	Galazzi et al,2018	I-1 nonno	M		-	-	-	-	-	-	n	-	-	-	-	-	-	+	-	-	-	-	-	-	+
					II-2 madre	F		-	+	-	-	-	+	+	-	-	-	+ (right)	-	-	+	-	-	+	+	-	-	+
					III-1 proband	M	14y	+	-	-	-	-		+	-	-	+	-	-	-	-	-	-	+	+	-	-	+
4	c.817A>T	p.Lys273Ter	truncating	Sasaki et al,2002	proband	M		-	-	+	-	-		+	-	-	+	+ (bilateral)	-	-	+	-	-	-	-	-	-	+
					brother	M		-	-	-	-	-		+	-	-	+	-	-	-	+	-	-	-	-	-	-	-
					mother	F	48y	-	-	-	-	-	+	+	-	-	-	-	-	-	+	-	+	+	-	-	-	-
3	c.804 + 1G > A	-	splicing	Tung et al,2022	father	M		-	-	-	-	+		+	-	-	-	-	-	-	+	-	-	-	-	+	-	-
					Twin 1	M	3 weeks	-	-	-	-	-	-	n	-	-	-	-	-	-	-	-	-	-	-	-	-	-
					Twin 2	M	3 weeks	-	-	-	-	-	-	n	-	-	-	-	-	-	+	-	-	-	-	-	-	-
					proband	F	1y	-	-	-	-	-	-	n	-	-	-	-	-	-	+	-	-	-	-	+	-	-
3	c.723delG	p.Lys242AsnfsTer20	truncating	Shelagh Joss et al. 2011	Twin	M	6y	-	+	-	-	+		+	+	-	+	+	+	+	+	+	-	-	-	-	-	-
2	c.657+1G>C	-	splicing	Bamshad M et al,1999	1	n		-	-	-	-	-	+	n	-	-	-	-	-	-	+	-	-	+	+	-	-	-
					2	n		-	-	-	-	-	-	n	-	-	+	-	-	-	+	-	-	+	+	-	-	+
2	c.570C>A	p.Ser190Arg	missense	Galazzi et al, 2018	I-2	F		-	-	-	-	-	-	n	-	-	-	-	-	-	-	-	-	-	+	-	-	-
					II-2	M		-	-	-	-	-		+	-	-	-	-	-	-	-	-	-	-	+	-	-	-
					III-2	M	59y	-	-	-	-	-		+	-	-	-	+	-	-	+	-	-	+	+	-	-	+
					IV-1	F	20y	-	-	-	-	-	+	+	-	-	-	-	-	-	-	-	-	+	+	-	-	+
					IV-2	M	14y	-	-	-	-	-		+	-	-	-	-	-	-	-	-	-	+	+	-	-	+
2	c.452_465dup	p.Ile156TyrfsTer2	truncating	Bamshad M et al,1999	1	M		-	-	-	-	+		+	-	-	-	-	-	-	+	-	-	+	+	-	-	+
					2	M		-	-	-	-	+		+	-	-	-	-	-	-	-	-	-	+	+	-	-	+
					3	M		-	-	+	-	+		+	-	-	+	-	-	-	+	-	-	+	+	-	-	+
					4	M		-	-	-	-	-		+	-	-	-	-	-	-	+	-	-	+	+	-	-	+
					5	M		-	-	-	-	-		+	-	-	-	-	-	-	+	-	-	+	+	-	-	+
					6	F		-	-	-	-	-	+	+	-	+	-	-	-	-	+	-	-	+	+	-	-	+
2	c.446A>C	p.Tyr149Ser	missense	Bamshad M et al,1999	1	M		-	-	-	-	-		+	-	-	-	-	-	-	+	-	-	+	+	+	-	+
2	c.428T>C	p.Leu143Pro	missense	Bamshad M et al,1999	1	M		-	-	-	-	-	-	n	-	-	-	-	-	-	-	-	-	+	+	-	-	+
					2	M		-	-	-	-	-		+	-	-	+	+	-	+	+	-	-	+	+	-	-	+
					3	M		-	-	-	-	-		+	-	-	+	-	-	-	+	-	-	+	+	-	-	+
					4	M		-	-	-	-	-		+	-	-	+	+	-	+	+	-	-	+	+	-	-	+
					5	F		-	-	-	-	-	+	+	-	-	-	+ (left)	-	+ (left)	+	-	-	+	+	-	-	+
2	c.418T>C	p.Cys140Arg	missense	ClinVar				n	n	n	n	n	n	n	n	n	n	n	n	n	n	n	n	n	n	n	n	n
**2**	**c.400C>T**	**p.Pro134Ser**	**missense**	**This report**	**mother**	**F**	**33y**	**-**	**-**	**+**	**-**	**-**	**+**	**n**	**-**	**+**	**-**	**-**	**-**	**-**	**+**	**-**	**-**	**-**	**-**	**-**	**-**	**-**
1	c.370G>T	p.Val124Phe	missense	ClinVar				n	n	n	n	n	n	n	n	n	n	n	n	n	n	n	n	n	n	n	n	n
1	c.337T>G	p.Trp113Gly	missense	ClinVar				n	n	n	n	n	n	n	n	n	n	n	n	n	n	n	n	n	n	n	n	n
1	c.227delT	p.Ile76ThrfsTer12	truncating	Bamshad M et al,1999	1	M		-	-	+	-	-	-	n	-	-	-	-	-	-	-	-	-	+	+	-	-	-
					2	F		+	-	+	-	-	+	+	-	-	-	-	-	-	+	-	-	+	+	-	-	-
					3	F		-	-	+	-	-	-	-	-	-	-	-	-	-	+	-	-	+	+	-	-	-
					4	M		+	-	+	-	-	+	+	+	-	+	-	-	-	+	-	-	+	+	-	-	+
1	c.88dupA	p.Met30AsnfsTer81	truncating	Wollnik B et al,2002	III-4	F		-	-	-	-	-	+	+	-	-	-	-	-	-	+	-	-	+	+	-	-	-
					IV-3	F		-	-	-	-	-	+	+	-	-	-	-	-	-	+	-	-	+	+	-	-	-
					IV-4	F		-	-	-	-	-	-	-	-	-	-	-	-	-	-	-	-	+	+	-	-	-
					IV-6	F		-	-	-	-	-	+	+	-	-	-	-	-	-	-	-	-	+	+	-	-	-
					IV-10	M		-	-	-	-	-	n	n	-	-	n	-	-	-	+	-	-	n	n	-	-	-
					V-5	F	7y	-	-	+	-	+	+	+	-	-	-	-	-	-	+	-	-	+	-	+	-	-
					V-6	M		-	+	+	-	-	-	+	-	-	+	-	-	-	+	+	-	+	-	-	-	-
					V-7	M		-	-	-	-	-	+	+	-	-	-	-	-	-	-	-	-	-	+	-	-	-
					V-8	M		-	-	-	-	-	+	+	-	-	-	-	-	-	+	-	-	-	-	-	-	-
					V-9	M		-	-	-	-	-	n	n	-	-	n	-	-	-	+	+	-	n	n	-	-	-

Supplementary Material 1 . Data collected for the clinical and molecular revision of the literature.
