# Supplementary material for: An inherited TBX3 alteration in a prenatal case of ulnar‐mammary syndrome: Clinical assessment and functional characterization in Drosophila melanogaster
Source: J Cell Physiol. 2024 Sep 25;239(12):e31440. doi: 10.1002/jcp.31440 (PMC11649972; doi:10.1002/jcp.31440)
Supplement: Supplementary file 3 — Supporting information. [file JCP-239-0-s002.pdf]

TBX3 CDS and translation

```
  1 ATGAGCCTCTCCATGAGAGATCCGGTCATTCCTGGGACAAGCATG
      M  S  L  S  M  R  D  P  V  I  P  G  T  S  M

 46 GCCTACCATCCGTTCCTACCTCACCGGGCGCCGGACTTCGCCATG
      A  Y  H  P  F  L  P  H  R  A  P  D  F  A  M

 91 AGCGCGGTGCTGGGTCACCAGCCGCCGTTCTTCCCCGCGCTGACG
      S  A  V  L  G  H  Q  P  P  F  F  P  A  L  T

136 CTGCCTCCCAACGGCGCGGCGGCGCTCTCGCTGCCGGGCGCCCTG
      L  P  P  N  G  A  A  A  L  S  L  P  G  A  L

181 GCCAAGCCGATCATGGATCAATTGGTGGGGGCGGCCGAGACCGGC
      A  K  P  I  M  D  Q  L  V  G  A  A  E  T  G

226 ATCCCGTTCTCCTCCCTGGGGCCCCAGGCGCATCTGAGGCCTTTG
      I  P  F  S  S  L  G  P  Q  A  H  L  R  P  L

271 AAGACCATGGAGCCCGAAGAAGAGGTGGAGGACGACCCCAAGGTG
      K  T  M  E  P  E  E  E  V  E  D  D  P  K  V

316 CACCTGGAGGCTAAAGAACTTTGGGATCAGTTTCACAAGCGGGGC
      H  L  E  A  K  E  L  W  D  Q  F  H  K  R  G
```

T

361 ACCGAGATGGTCATTACCAAGTCGGGAAGGCGAATGTTTCCTCCA

T E M V I T K S G R R M F P P

S **P134S**

406 TTTAAAGTGAGATGTTCTGGGCTGGATAAAAAAGCCAAATACATT

F K V R C S G L D K K A K Y I

451 TTATTGATGGACATTATAGCTGCTGATGACTGTCGTTATAAATTT

L L M D I I A A D D C R Y K F

496 CACAATTCTCGGTGGATGGTGGCTGGTAAGGCCGACCCCGAAATG

H N S R W M V A G K A D P E M

541 CCAAAGAGGATGTACATTCACCCGGACAGCCCCGCTACTGGGGAA

P K R M Y I H P D S P A T G E

586 CAGTGGATGTCCAAAGTCGTCACTTTCCACAAACTGAAACTCACC

Q W M S K V V T F H K L K L T

631 AACAACATTTCAGACAAACATGGATTTACTATATTGAACTCCATG

N N I S D K H G F T I L N S M

676 CACAAATACCAGCCCCGGTTCCACATTGTAAGAGCCAATGACATC

H K Y Q P R F H I V R A N D I

721 TTGAAACTCCCTTATAGTACATTTCGGACATACTTGTTCCCCGAA

```
      L  K  L  P  Y  S  T  F  R  T  Y  L  F  P  E

766  ACTGAATTCATCGCTGTGACTGCATACCAGAATGATAAGATAACC
      T  E  F  I  A  V  T  A  Y  Q  N  D  K  I  T

                           G
811  CAGTTAAAAATAGACAACAACCCTTTTGCAAAAGGTTTCCGGGAC
      Q  L  K  I  D  N  N  P  F  A  K  G  F  R  D      N277S
                        S

856  ACTGGAAATGGCCGAAGAGAAAAAAGAAAACAGCTCACCCTGCAG
      T  G  N  G  R  R  E  K  R  K  Q  L  T  L  Q

901  TCCATGAGGGTGTTTGATGAAAGACACAAAAAGGAGAATGGGACC
      S  M  R  V  F  D  E  R  H  K  K  E  N  G  T

946  TCTGATGAGTCCTCCAGTGAACAAGCAGCTTTCAACTGCTTCGCC
      S  D  E  S  S  S  E  Q  A  A  F  N  C  F  A

991  CAGGCTTCTTCTCCAGCCGCCTCCACTGTAGGGACATCGAACCTC
      Q  A  S  S  P  A  A  S  T  V  G  T  S  N  L

1036 AAAGATTTATGTCCCAGCGAGGGTGAGAGCGACGCCGAGGCCGAG
      K  D  L  C  P  S  E  G  E  S  D  A  E  A  E

1081 AGCAAAGAGGAGCATGGCCCCGAGGCCTGCGACGCGGCCAAGATC
      S  K  E  E  H  G  P  E  A  C  D  A  A  K  I
```

1126 TCCACCACCACGTCGGAGGAGCCCTGCCGTGACAAGGGCAGCCCC

S T T T S E E P C R D K G S P

1171 GCGGTCAAGGCTCACCTTTTCGCTGCTGAGCGGCCCCGGGACAGC

A V K A H L F A A E R P R D S

1216 GGGCGGCTGGACAAAGCGTCGCCCGACTCACGCCATAGCCCCGCC

G R L D K A S P D S R H S P A

1261 ACCATCTCGTCCAGCACTCGCGGCCTGGGCGCGGAGGAGCGCAGG

T I S S S T R G L G A E E R R

1306 AGCCCGGTTCGCGAGGGCACAGCGCCGGCCAAGGTGGAAGAGGCG

S P V R E G T A P A K V E E A

1351 CGCGCGCTCCCGGGCAAGGAGGCCTTCGCGCCGCTCACGGTGCAG

R A L P G K E A F A P L T V Q

1396 ACGGACGCGGCCGCCGCGCACCTGGCCCAGGGCCCCCTGCCTGGC

T D A A A A H L A Q G P L P G

1441 CTCGGCTTCGCCCCGGGCCTGGCGGGCCAACAGTTCTTCAACGGG

L G F A P G L A G Q Q F F N G

1486 CACCCGCTCTTCCTGCACCCCAGCCAGTTTGCCATGGGGGGCGCC

H P L F L H P S Q F A M G G A

1531 TTCTCCAGCATGGCGGCCGCTGGCATGGGTCCCCTCCTGGCCACG

F S S M A A A G M G P L L A T

T

1576 GTTTCTGGGGCCTCCACCGGTGTCTCGGGCCTGGATTCCACGGCC

V S G A S T G V S G L D S T A **T539M**

M

1621 ATGGCCTCTGCCGCTGCGGCGCAGGGACTGTCCGGGGCGTCCGCG

M A S A A A A Q G L S G A S A

1666 GCCACCCTGCCCTTCCACCTCCAGCAGCACGTCCTGGCCTCTCAG

A T L P F H L Q Q H V L A S Q

1711 GGCCTGGCCATGTCCCCTTTCGGAAGCCTGTTCCCTTACCCCTAC

G L A M S P F G S L F P Y P Y

1756 ACGTACATGGCCGCAGCGGCGGCCGCCTCCTCTGCGGCAGCCTCC

T Y M A A A A A A S S A A A S

1801 AGCTCGGTGCACCGCCACCCCTTCCTCAATCTGAACACCATGCGC

S S V H R H P F L N L N T M R

1846 CCGCGGCTGCGCTACAGCCCCTACTCCATCCCGGTGCCGGTCCCG

P R L R Y S P Y S I P V P V P

1891 GACGGCAGCAGTCTGCTCACCACCGCCCTGCCCTCCATGGCGGCG

```
        D  G  S  S  L  L  T  T  A  L  P  S  M  A  A

1936 GCCGCGGGGCCCCTGGACGGCAAAGTCGCCGCCCTGGCCGCCAGC
        A  A  G  P  L  D  G  K  V  A  A  L  A  A  S

1981 CCGGCCTCGGTGGCAGTGGACTCGGGCTCTGAACTCAACAGCCGC
        P  A  S  V  A  V  D  S  G  S  E  L  N  S  R

2026 TCCTCCACGCTCTCCTCCAGCTCCATGTCCTTGTCGCCCAAACTC
        S  S  T  L  S  S  S  S  M  S  L  S  P  K  L

                     A
                     v
2071 TGCGCGGAGAAAGAGGCGGCCACCAGCGAACTGCAGAGCATCCAG
        C  A  E  K  E  A  A  T  S  E  L  Q  S  I  Q
                    R  G  G  H  Q  R  T  A  E  H  P     E695R

2116 CGGTTGGTTAGCGGCTTGGAAGCCAAGCCGGACAGGTCCCGCAGC
        R  L  V  S  G  L  E  A  K  P  D  R  S  R  S
        A  V  G  *

2161 GCGTCCCCGTAG
        A  S  P  *
```

Supplementary Material 2. Position of the four point variants (with the corresponding amino acid changes in the TBX protein), that have been introduced in the TBX CDS and cloned in the *pUAS-attB* vector (see also Materials and Methods).
